# Solvent-Dependent Selective Retention of Essential-Oil Markers and Modification of the Thymol-Type Chemical Fingerprint in Post-Extraction *Thymus vulgaris* L. Biomass

**DOI:** 10.3390/molecules31213796

**Published:** 2026-10-22

**Authors:** Radosław Kowalski, Klaudia Kałwa, Artur Mazurek, Grażyna Kowalska

**Affiliations:** 1 Department of Analysis and Evaluation of Food Quality, University of Life Sciences in Lublin, 20-704 Lublin, Poland; artur.mazurek@up.edu.pl; 2 Department of Dietetics, Institute of Human Nutrition and Agriculture Sciences, The State University of Applied Sciences in Chełm, 22-100 Chełm, Poland; klaudia.kalwa91@gmail.com; 3 Department of Tourism and Recreation, University of Life Sciences in Lublin, 20-950 Lublin, Poland; grazyna.kowalska@up.edu.pl

**Keywords:** *Thymus vulgaris*, essential oil, thymol, p-cymene, γ-terpinene, post-extraction biomass, selective retention, chemical fingerprint, compositional data analysis, green extraction

## Abstract

This study evaluated whether short-term extraction of *Thymus vulgaris* L. with solvents of different polarity selectively modifies the essential-oil (EO) fingerprint of post-extraction biomass. The initial material contained 1.85% *v*/*w* EO, dominated by thymol (53.65%), p-cymene (21.53%), and γ-terpinene (5.28%). Water, 65% glycerol–water, rapeseed oil, and 50% ethanol–water were compared under different time and temperature conditions. Selective retention of six markers was assessed using an apparent marker retention index (AMRI), combining residual EO content with relative GC-FID composition, together with SEF, thymol-profile ratios, and CLR-PCA. Under solvent-specific reference conditions, thymol AMRI was 59.2% for water, 39.3% for glycerol, 18.4% for oil, and 27.2% for ethanol. The strongest relative enrichment occurred for (E)-caryophyllene after glycerol extraction (AMRI 149.8%; SEF 3.26). CLR-PCA explained 90.2% of compositional variability in the first two components and revealed solvent-dependent reorganization of the marker profile. Water and nanowater produced the highest integrated polar-functional response based on TPC, TFC, and DPPH. These results show that extraction can selectively modify the chemical fingerprint of the residual volatile fraction without implying a change in the biologically determined chemotype, supporting targeted fractionation and sequential use of post-extraction thyme biomass.

## 1. Introduction

Common thyme (*Thymus vulgaris* L.) is one of the most important aromatic species of the Lamiaceae family and has been used for centuries both as a culinary herb and as a herbal raw material. Its importance arises from the combination of an intense aroma and a high content of secondary metabolites with technological and biological properties. In foods, thyme is used not only as a seasoning but also as a potential source of natural preservatives and antioxidants. Experimental studies have shown that the essential oil and extracts of *T. vulgaris* exhibit antioxidant and antimicrobial activity [1,2]. The EMA monograph also recognizes the traditional use of thyme herb as an expectorant in cough associated with colds [3]. From a food technology perspective, two groups of constituents are particularly relevant: the volatile fraction, represented by the essential oil, and the polar fraction, which includes phenolic compounds and flavonoids.

The phytochemical profile of *T. vulgaris* is complex. Its essential oil may be dominated by thymol, carvacrol, p-cymene, γ-terpinene, linalool, and other mono- and sesquiterpenes [1,2,4,5]. The non-volatile fraction contains, among others, rosmarinic, chlorogenic, and caffeic acids, as well as luteolin and apigenin derivatives [6]. The coexistence of volatile and polar fractions makes it difficult to assess raw-material quality or design an extraction process on the basis of a single parameter. Total phenolic content or antioxidant activity may characterize the potential of an aqueous or hydrophilic extract, but neither describes the behavior of the essential oil. Conversely, EO content alone does not show whether key marker compounds and the proportions characteristic of the original material are retained after processing.

A distinctive feature of thyme is the considerable variability in essential-oil composition. Thymol, carvacrol, linalool, geraniol, borneol, and other chemotypes have been described in *T. vulgaris*, and their occurrence depends on genetic background, geographical origin, environmental conditions, and developmental stage [4,7,8]. The observed essential-oil profile may be further modified by harvesting and processing conditions [9,10]. In the thymol-type profile, the γ-terpinene–p-cymene–thymol system is particularly important. Classical studies on monoterpene biosynthesis in *T. vulgaris* have shown that these compounds are metabolically related, with γ-terpinene and p-cymene forming part of the pathway leading to thymol [11]. Examining the relationships among these constituents may therefore be more informative than considering thymol alone. A biologically determined plant chemotype should also be distinguished from changes in essential-oil composition caused by technological processing. Drying, grinding, and granulation have been shown to affect both total EO content and the relative proportions of its constituents [9,10], indicating that processing may preserve, distort, or selectively deplete the chemical fingerprint of the material.

Extraction represents a further stage of such technological fractionation. Its outcome depends on solvent polarity and viscosity, the phase affinity of individual compounds, temperature, contact time, and the properties of the plant matrix. Aqueous systems favor the release of polar compounds, whereas hydroalcoholic or lipid phases may have a greater affinity for volatile and lipophilic constituents. Glycerol–water occupies an intermediate position: it is food-grade, non-volatile, and miscible with water, but its viscosity may limit mass-transfer rates, particularly at lower temperatures [12,13,14]. Extraction time and temperature should thus be considered in relation to the properties of the solvent and the group of metabolites being recovered rather than as universally optimal parameters. Studies on Lamiaceae plants also indicate that material remaining after the first extraction step may still contain substantial amounts of phenolic compounds and volatile constituents, which provides a rationale for secondary extraction and for the potential valorization of post-extraction biomass [15,16].

Current approaches to the recovery of plant-derived constituents increasingly combine extraction efficiency with green-chemistry principles, reduced energy use, and the possibility of using the resulting extracts directly in foods or supplements [17]. In this context, simple systems based on water, glycerol–water and ethanol–water mixtures, nanowater, or vegetable oils are of interest because they can operate under conditions close to those used for preparing herbal infusions and do not require intensive purification or solvent evaporation [12,13,15,16,18]. Nanowater was included in the present study as an exploratory water-based medium to determine whether a commercially available plasma-treated water would produce a different overall TPC/TFC/DPPH response from distilled water under comparable extraction conditions. This comparison was motivated by previous evidence that physicochemical characteristics of water, including mineralization, can influence the extraction of phenolic compounds and the antioxidant properties of herbal infusions [18]. No specific mechanism related to plasma treatment was assumed. In a separate earlier study on *T. vulgaris*, simple low-processed extraction systems were shown to recover a substantial proportion of TPC and TFC from the raw material and to provide an alternative to highly concentrated commercial extracts [19].

That study, however, focused on the recovery of polar compounds and on comparisons between low- and highly processed extracts; it did not examine the selective retention of individual essential-oil markers in post-extraction material or quantitative–compositional shifts in the thymol-type profile. The earlier study on peppermint [16] used a related extraction scheme but involved a different plant material and a different analytical scope. The novelty of the present study lies in combining the measured residual EO content with a six-marker GC-FID profile and in evaluating changes in apparent marker retention and the compositional structure of the post-extraction thyme material.

Studies on sequential extraction of herbal materials indicate that solvent type can differentiate the recovery of polar and volatile fractions, while the material remaining after the first extraction step may retain constituents that can be recovered or used subsequently [15,16]. This issue is particularly relevant for thyme because of its distinct chemotype-related EO marker pattern. Knowing only how much essential oil remains after extraction does not show whether thymol, p-cymene, γ-terpinene, linalool, and sesquiterpenes retain proportions similar to those in the starting material. Likewise, an increase in the percentage contribution of a constituent in a normalized chromatogram does not necessarily indicate a greater amount of that compound in the material, as it may simply result from lower retention of other constituents. Interpretation thus requires the EO balance to be considered together with the GC profile and the response of the polar fraction.

The aim of this study was to investigate the solvent-dependent selective retention of six thyme essential-oil markers—myrcene, p-cymene, γ-terpinene, linalool, thymol, and (E)-caryophyllene—in plant material after primary and secondary extraction. For this purpose, relative GC composition was combined with the directly measured amount of essential oil remaining in the material, and an apparent marker retention index (AMRI) was introduced together with complementary indices describing relative enrichment and thymol-profile ratios. In parallel, TPC, TFC, and DPPH results were used for a multiresponse assessment of the polar-functional fraction as a function of extraction time, temperature, and solvent type. We hypothesized that solvents differing in polarity and phase affinity would not only alter EO content quantitatively but would also selectively modify the chemical fingerprint of the remaining volatile fraction, and that the direction of this modification could differ from that favoring the recovery of polar compounds. From this perspective, extraction can be viewed not as a route to a single “best” extract, but as a means of targeted fractionation and sequential use of different bioactive fractions of the plant material.

## 2. Results and Discussion

### 2.1. Characteristics of the Initial Material and the Thymol-Type Profile

The essential-oil data are summarized in Supplementary Tables S5–S8. The EO content of thyme herb was 1.85% *v*/*w*. Forty-six compounds were identified, accounting for 99.44% of the total profile. Thymol was the dominant constituent (53.65%), followed by p-cymene (21.53%), γ-terpinene (5.28%), carvacrol (3.30%), linalool (1.90%), and (E)-caryophyllene (1.50%). This composition clearly corresponds to a thymol-type profile. It also falls within the range reported for *T. vulgaris*: Soković et al. [20] found 48.9% thymol and 19.0% p-cymene, whereas Galovičová et al. [2] reported 48.1% thymol, 11.7% p-cymene, 6.1% γ-terpinene, and 5.5% carvacrol in a commercial thymol-type oil. Population- and chemotype-based studies have nevertheless shown that the proportions of these constituents may vary widely depending on the origin and type of plant material [4,8,21].

All experimental variants in the present study originated from the same homogenized batch, so the differences observed after extraction were not attributable to between-batch variability. This is particularly important when interpreting the chemotype-related profile. A short technological process may modify the recovered EO fingerprint through selective transfer and retention of individual constituents without, by itself, providing evidence of a change in the biologically determined chemotype. Similar sensitivity of thymol, p-cymene, and γ-terpinene proportions to processing conditions has been reported in studies on thyme drying [9,22]. The six constituents selected for marker analysis and their roles in the subsequent analysis are summarized in Table 1.

### 2.2. Essential-Oil Balance and the Potential of Post-Extraction Material

**Figure 1:**
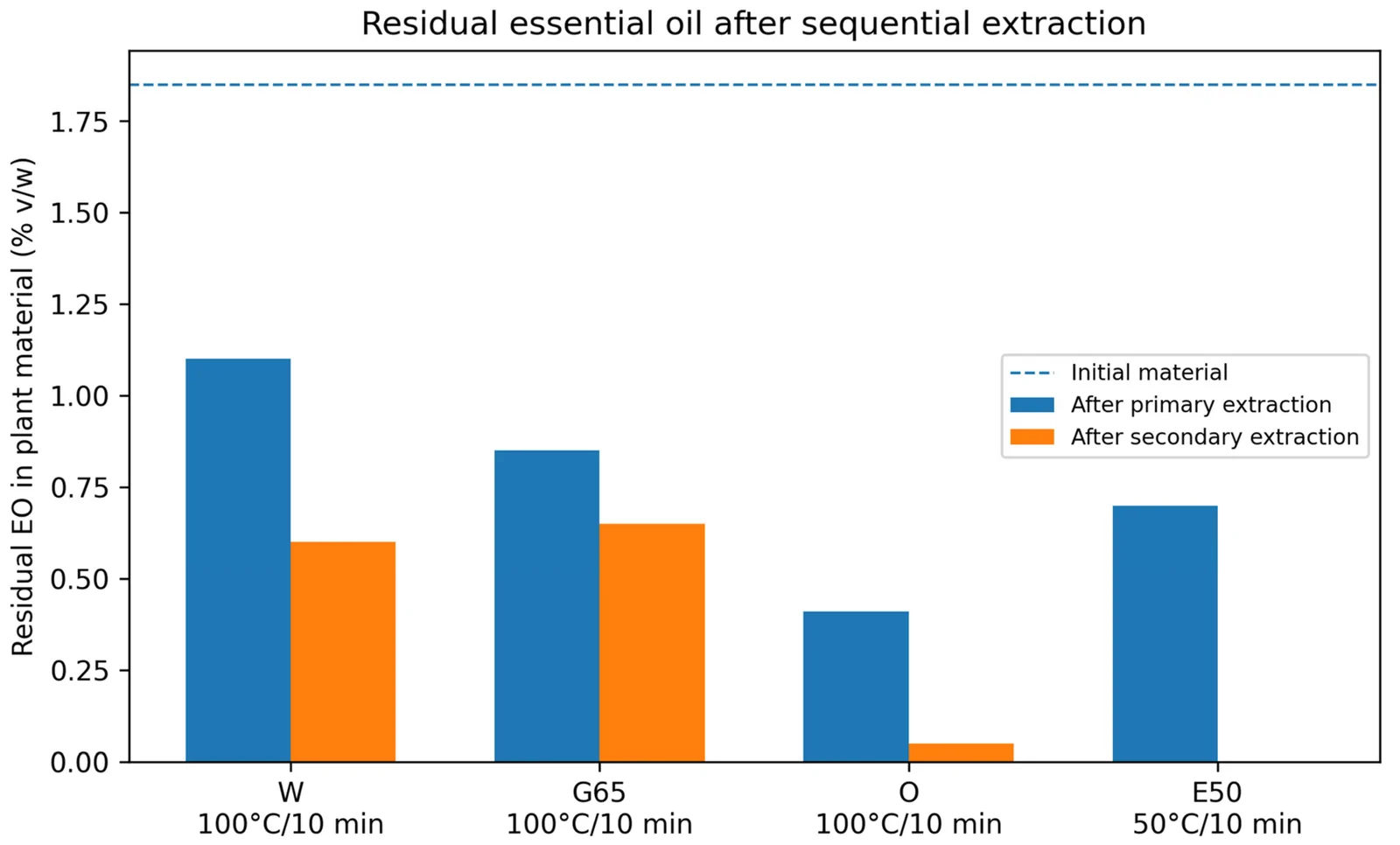
Residual essential oil in the initial plant material and after primary and secondary extraction at the solvent-specific reference conditions (W, G65 and O: 100 °C/10 min; E50: 50 °C/10 min). Values are based on direct EO determinations in plant material. Solvent codes: W—water; G65—65% glycerol–water; O—rapeseed oil; E50—50% ethanol–water.

Changes in EO content after extraction depended strongly on the extraction medium. Under the reference conditions, EO content decreased from 1.85% *v*/*w* in the initial material to 1.10% after water extraction (100 °C/10 min), 0.85% after extraction with 65% glycerol (100 °C/10 min), 0.41% after extraction with rapeseed oil (100 °C/10 min), and 0.70% after extraction with 50% ethanol (50 °C/10 min). The relative decrease in EO recoverable from the biomass (ΔEO_rel_) was 40.5%, 54.1%, 77.8%, and 62.2%, respectively. This value should not be interpreted as the amount of essential oil transferred to the liquid phase. After the second extraction step, the residual EO contents were 0.60%, 0.65%, 0.05%, and 0.00% *v*/*w* (not volumetrically measurable) for W, G65, O, and E50, respectively. These results point to two distinct process patterns (Figure 1): after water and glycerol extraction, the biomass retained a substantial proportion of the oil fraction, whereas EO content declined much more strongly in the oil and ethanol–water systems. The complete primary and secondary EO balance across the tested conditions is provided in Supplementary Table S5.

These differences may be related to differences in phase affinity of the volatile constituents toward the extraction media. The presence of a measurable EO fraction after the first extraction step also indicates that the post-extraction material may still have value for further processing. The concept of sequential valorization of aromatic biomass is supported by studies on cascade utilization of essential-oil plants [23] and on Lamiaceae by-products, including thyme, in which valuable bioactive fractions remained detectable after preceding processing steps [24]. This is consistent with viewing post-extraction material as a potential secondary raw material rather than as a final waste stream [15].

### 2.3. Selective Retention of Markers After Primary Extraction

Under the reference conditions, the EO profile after extraction differed clearly among the media. After water extraction, the proportion of thymol remained close to that in the initial material (53.40 vs. 53.65%), whereas after G65, O, and E50 extraction it decreased to 45.92, 44.59, and 38.60%, respectively. G65 and O increased the relative proportions of γ-terpinene and (E)-caryophyllene, while E50 extraction produced a marked increase in the proportion of linalool. These shifts indicate constituent-specific effects of the process and justify considering the GC profile together with the balance of the total EO fraction.

The complete six-marker GC-FID profiles after primary extraction are provided in Supplementary Table S6, whereas the corresponding AMRI and SEF calculations at the reference conditions are given in Supplementary Table S8.

Because GC-FID area% is a relative measure, an increase in area% does not automatically indicate a larger amount of a given compound in the plant material. When EO content was taken into account, thymol AMRI was highest after water extraction (59.2% of the initial marker load), followed by glycerol (39.3%), ethanol (27.2%), and oil extraction (18.4%). p-Cymene showed lower retention than thymol in each of these systems, with AMRI values of 42.5% for water, 20.1% for glycerol, 14.4% for oil, and 16.0% for ethanol. A different pattern was observed for γ-terpinene, which was relatively well retained after glycerol (76.1%) and water extraction (69.6%), and for linalool, for which the highest AMRI was found after ethanol extraction (82.0%). Selected AMRI and SEF values for the 10 min reference conditions are summarized in Table 2.

**Figure 2:**
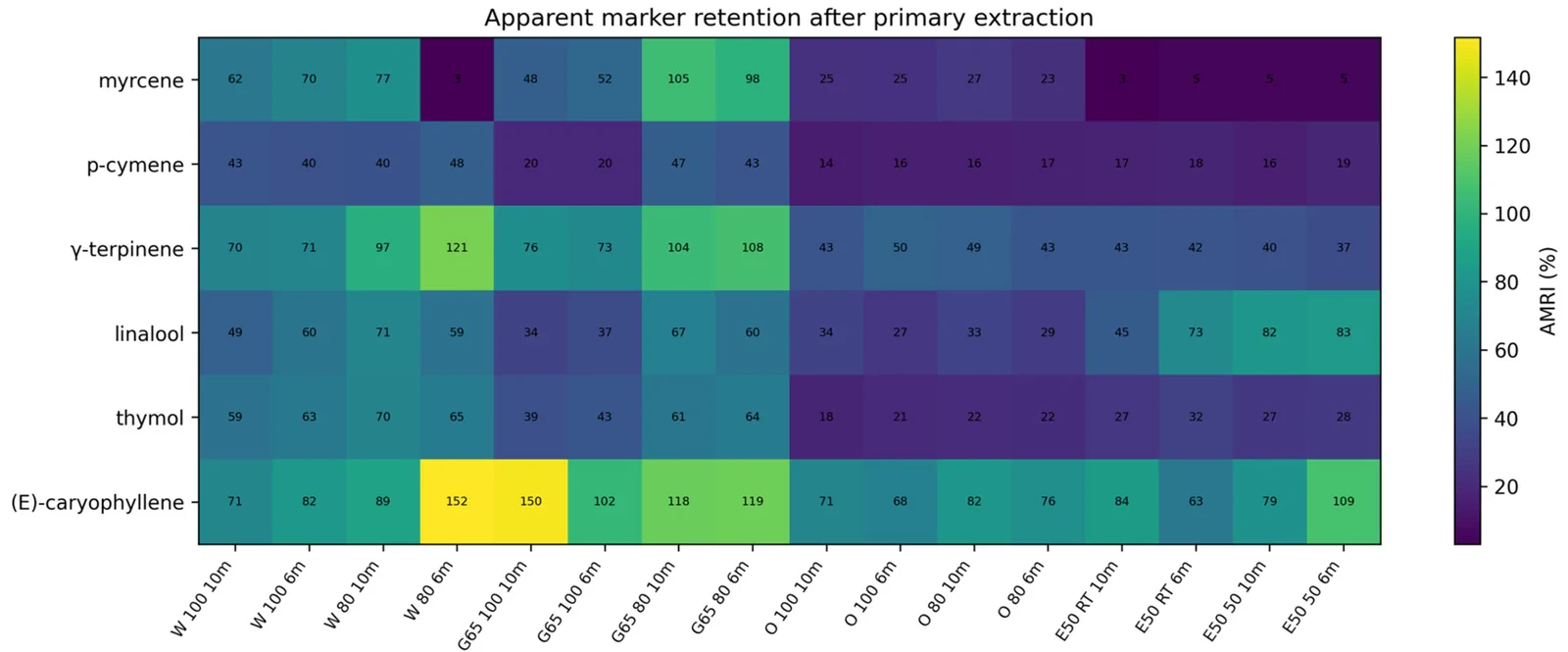
Heatmap of the apparent marker retention index (AMRI) after primary extraction. Numbers in the cells denote AMRI (%). Solvent codes: W—water; G65—65% glycerol–water; O—rapeseed oil; E50—50% ethanol–water; RT—room temperature.

The strongest relative enrichment was observed for (E)-caryophyllene. After glycerol extraction, its proportion increased from 1.50 to 4.89%, while the derived AMRI was 149.8%. This is an operational value based on the volume of recovered EO and the relative peak area, rather than evidence of an increase in the absolute mass of (E)-caryophyllene. Similar shifts in the proportions of monoterpenes and sesquiterpenes, depending on the method of recovery or processing, have previously been reported for thyme oil [9,22,25]. Under different, subcritical conditions, Shin and Ko [26] also observed non-uniform responses among individual monoterpenes, with oxygenated monoterpenes being extracted more efficiently than non-oxygenated compounds. Their results support the interpretation that individual EO constituents need not respond proportionally to processing conditions. The broader pattern of AMRI values across the primary-extraction variants is shown in Figure 2. This result refers to the EO profile recovered under the applied procedure; the physicochemical properties and volatility of individual terpenes may also affect their relative recovery during hydrodistillation.

### 2.4. Modification of the Thymol-Type Fingerprint and Ratio-Based Indices

In the initial material, CBR was 2.00 and the thymol/p-cymene ratio was 2.49. After 10 min of primary extraction, CBR increased to 2.48 for water and 2.53 for glycerol, while values of 1.85 and 2.62 were obtained for oil and ethanol, respectively. This indicates that in the water, glycerol, and ethanol–water systems, p-cymene was retained to a lesser extent than thymol, whereas after oil extraction the relationship between thymol and the combined p-cymene + γ-terpinene pool shifted in the opposite direction. The thymol/p-cymene ratio after primary extraction was 3.47 (W), 4.88 (G65), 3.19 (O), and 4.23 (E50), whereas after secondary extraction it was 3.07 (W), 2.67 (G65), and 2.43 (O) (Table S9). After the second extraction step, the ratios moved closer to that observed in the initial material (2.49).

The γ-terpinene–p-cymene–thymol system is biosynthetically linked, and classical isotope studies demonstrated the sequence γ-terpinene → p-cymene → thymol [11]. In the present material, changes in CBR and the thymol/p-cymene ratio describe solvent-dependent shifts in the relative proportions of these compounds. This interpretation is also supported by results obtained from physicochemical fractionation of thyme essential oil using SAFE [27]. In the less volatile fraction, the relative proportions of γ-terpinene and p-cymene were markedly lower than in the original oil, whereas thymol and carvacrol increased. Such findings show that physicochemical fractionation alone can substantially shift the proportions within the γ-terpinene–p-cymene–thymol system.

### 2.5. Secondary Extraction and the Compositional Trajectory of Residual Material

The second extraction step further increased the differences among the systems. After secondary water extraction (100 °C/10 min), the material contained 0.60% *v*/*w* EO, with thymol accounting for 40.50%, p-cymene 13.20%, γ-terpinene 4.12%, linalool 1.02%, and (E)-caryophyllene 1.40%. These values corresponded to AMRI values of 24.5% for thymol and 19.9% for p-cymene relative to the initial material. After secondary extraction with 65% glycerol, 0.65% *v*/*w* EO remained, containing 46.50% thymol, 17.40% p-cymene, 5.70% γ-terpinene, 1.74% linalool, and 2.14% (E)-caryophyllene. The corresponding AMRI values were 30.5%, 28.4%, 37.9%, and 50.1% for thymol, p-cymene, γ-terpinene, and (E)-caryophyllene, respectively. After two extraction steps with oil, only 0.05% *v*/*w* EO remained; consequently, even relatively high proportions of individual constituents translated into AMRI values of only about 2–5% for the major markers.

Complete marker profiles after secondary extraction are provided in Supplementary Table S7, with the corresponding reference-condition AMRI and SEF values in Supplementary Table S8.

After secondary E50 extraction, no separate EO volume could be quantified volumetrically, although volatile constituents remained detectable in the xylene collection phase and were characterized by GC-FID as a relative compositional profile. Because no quantitative residual EO value was available, these profiles were excluded from AMRI calculations and CLR-PCA. This is relevant to cascade processing: similar approaches aimed at more complete use of aromatic biomass have been proposed for essential-oil crops [23], while studies on Lamiaceae by-products confirm that material remaining after an earlier processing step may retain valuable metabolites and functional properties [24].

### 2.6. Exploratory Compositional Analysis

**Figure 3:**
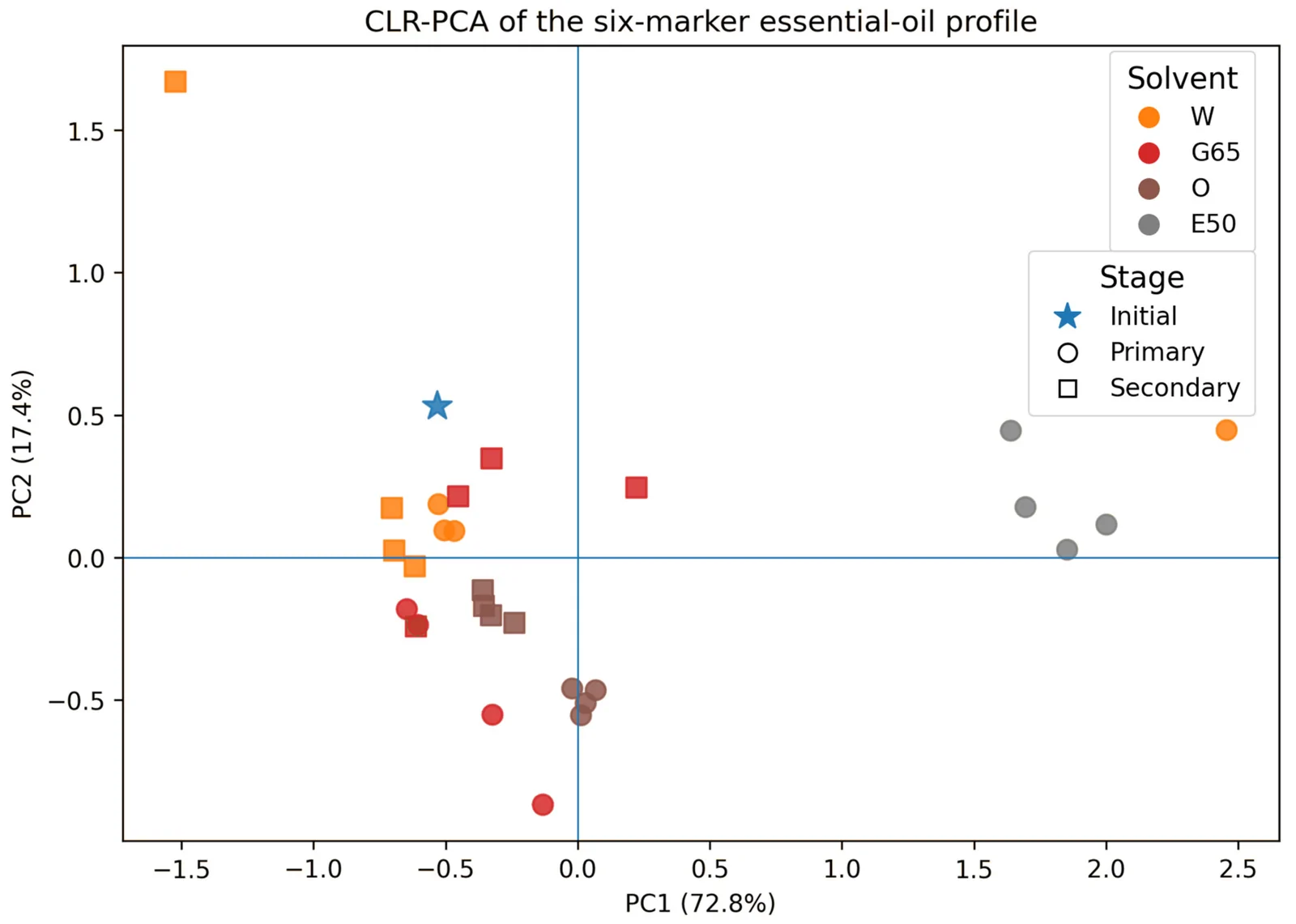
CLR-PCA score plot of the six-marker essential-oil profile of Thymus vulgaris before and after extraction. Colors denote solvent systems, whereas marker shapes denote extraction stage: star—initial material; circles—primary extraction; squares—secondary extraction. Solvent codes: W—water; G65—65% glycerol–water; O—rapeseed oil; E50—50% ethanol–water. PC1 and PC2 explain 72.8% and 17.4% of the total variance, respectively.

PCA performed after CLR transformation of the six markers (Figure 3) showed that PC1 explained 72.8% of the variability and PC2 17.4%, with the first two components together accounting for 90.2% of the compositional variance. Post-extraction profiles occupied different positions depending on the solvent and extraction stage. Inspection of the marker loadings (Table S14) showed that PC1 was driven primarily by myrcene (loading −0.863), with smaller contributions from (E)-caryophyllene (0.386) and linalool (0.289). PC2 was dominated by (E)-caryophyllene (−0.773), with p-cymene (0.427), thymol (0.306), and linalool (0.263) contributing in the opposite direction. The CLR-PCA was based on the six-marker GC-FID subcomposition and was intended as an exploratory analysis; distances between points in the score plot should not be interpreted as formal tests of statistical significance. Overall, the PCA indicates that extraction modified the relative relationships among several EO markers rather than affecting the thymol-type profile through changes in a single major constituent.

CLR transformation reduced the closure effect inherent in percentage data [28], allowing PCA to reflect mainly changes in the relationships among markers. The separation of profiles associated with different extraction media indicates that EO composition was modified in a multicomponent manner rather than through changes in a single dominant constituent.

### 2.7. Multivariate Response of the Polar Fraction and Its Relationship with the EO Balance

TPC, TFC, and DPPH activity were considered jointly as three complementary responses describing the polar-functional effect of extraction. Their joint evaluation allowed us to examine whether changes in phenolic and flavonoid concentrations occurred in parallel with DPPH scavenging capacity and how these relationships varied with extraction time, temperature, and solvent type. TPC, TFC, and DPPH represent overall spectrophotometric assay responses rather than quantitative measurements of individual metabolites. In the oil extract, the TPC response may also include the contribution of lipophilic phenolic compounds, including thymol transferred from the plant material into the oil phase; the response of the pure medium was accounted for in the analytical control.

TPC, TFC, and DPPH were measured in triplicate and analyzed statistically as described in the Methods; complete values, including SDs and Tukey groups, are provided in Supplementary Tables S1–S4. Log_2_ fold-change values, IPFI, correlations, and PCA were calculated from condition means and used as an additional descriptive and chemometric layer.

#### 2.7.1. Effects of Time and Temperature: Process Sensitivity Matrix

**Figure 4:**
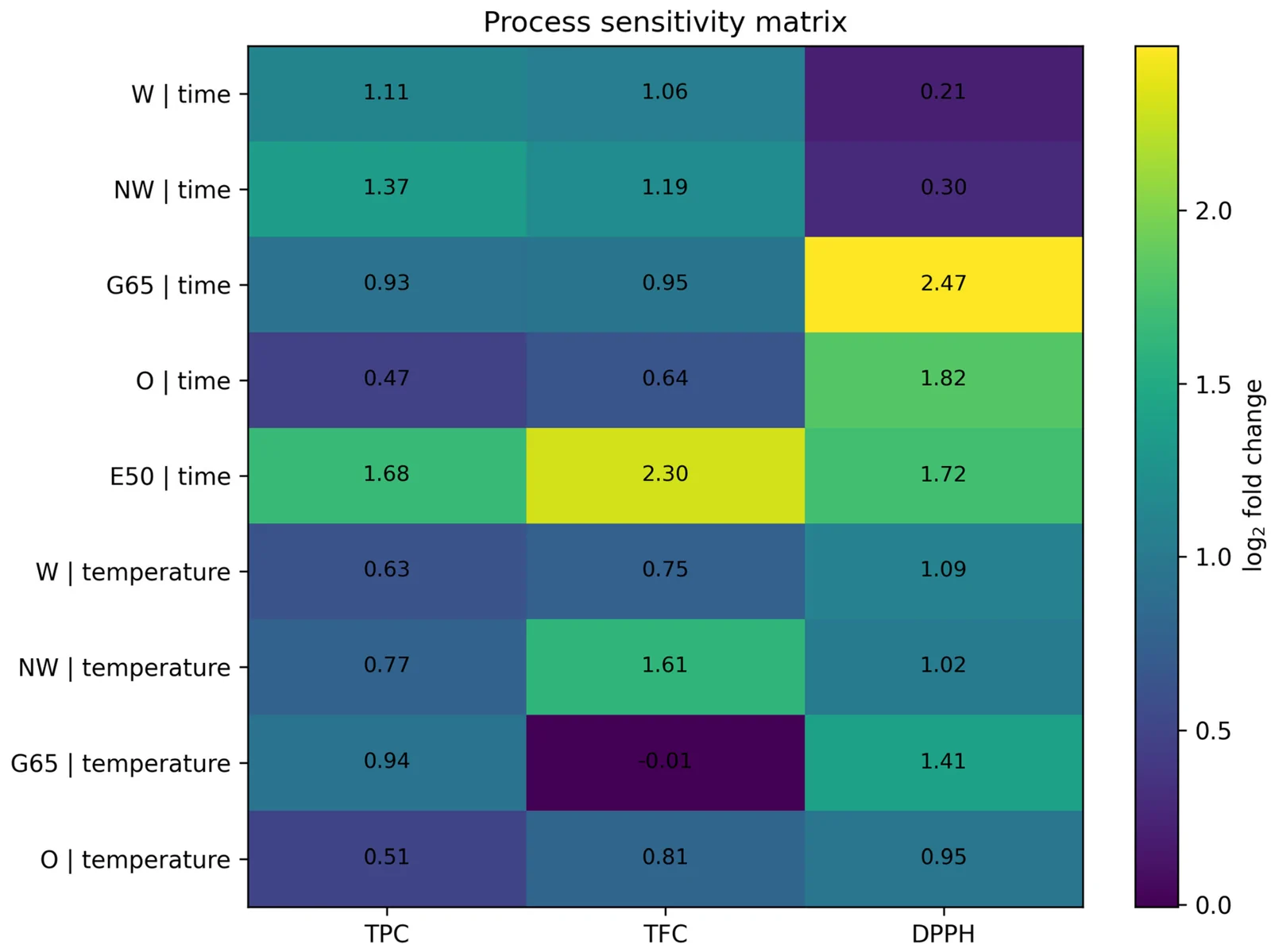
Process sensitivity matrix for TPC, TFC and DPPH. Values are log_2_ fold changes: 10 min versus 2 min for the time effect, and 100 °C versus 50 °C at 10 min for the temperature effect. E50 is shown only for the time series because its temperature scheme was not directly comparable with the other solvent systems.

The process sensitivity matrix (Figure 4) showed that extraction time did not affect all three responses to the same extent. When extraction was extended from 2 to 10 min, the largest increases in TPC and TFC occurred for E50 (1.68 and 2.30 log_2_, corresponding to approximately 3.20- and 4.92-fold increases) and NW (1.37 and 1.19 log_2_). In water, TPC and TFC increased approximately twofold (1.11 and 1.06 log_2_), whereas DPPH increased by only 0.21 log_2_. G65 and O showed the opposite pattern: changes in TPC and TFC were moderate, while the DPPH response increased by 2.47 and 1.82 log_2_, respectively, corresponding to approximately 5.56- and 3.53-fold increases. The increase in radical-scavenging capacity was thus not a simple function of total phenolic or flavonoid content. The log_2_ fold-change values used in the process sensitivity matrix are provided in Supplementary Table S11.

The effect of temperature also depended on both the extraction medium and the response considered. For W, increasing the temperature from 50 to 100 °C raised TPC, TFC, and DPPH by 0.63, 0.75, and 1.09 log_2_, respectively. In NW, TFC was particularly temperature-sensitive, increasing by 1.61 log_2_, or approximately 3.06-fold. For G65, TPC increased by 0.94 log_2_ and DPPH by 1.41 log_2_, whereas TFC remained essentially unchanged (−0.01 log_2_). Similar temperature dependence has been reported for water–glycerol systems, in which temperature significantly affected the kinetics and efficiency of polar-compound extraction [12,13]. Using pressurized hot-water extraction of deodorized thyme, Vergara-Salinas et al. [29] found that maximum recovery of many polyphenols was already reached at 100 °C after a short extraction time, whereas further increases in temperature and duration altered the phenolic profile without producing a linear relationship between phenolic content and antioxidant activity. A similar lack of complete parallelism between TPC/TFC and DPPH can be seen in our data, particularly for G65 and W. For NW, differences from distilled water may also be related to its physicochemical characteristics, including its nominal pH and conductivity. Previous work has shown that water mineralization can affect phenolic extraction and antioxidant response [18]; however, the mineral composition and other physicochemical properties of NW were not independently characterized in the present study. The E50 variant was compared with W, G65, and O under different initial-temperature conditions, so this comparison does not allow the effect of the extraction medium to be separated from that of the temperature protocol.

The proposed procedure was designed to evaluate low-processed extracts obtained from coarsely ground herb rather than the maximum yield achievable by exhaustive extraction of powdered plant material. For E50, TPC, TFC, and DPPH values increased between 2 and 10 min, indicating that the outcome depended on the short contact time. Differences in hydration and release kinetics in 50% ethanol may have contributed to this response, although this mechanism was not directly investigated. The process was deliberately not intensified by further grinding the material to a powder or by using assisted techniques such as ultrasound, because the aim was to evaluate a simple, low-processed extraction system while also assessing the residual essential-oil fraction. The results therefore describe the performance of a defined technological protocol rather than the general capacity of 50% ethanol to extract polyphenols.

#### 2.7.2. Multivariate TPC–TFC–DPPH Trajectories

PCA of the time series (Figure S1) explained 99.1% of the total variability in the first two components (PC1 = 90.9%; PC2 = 8.2%). Similar positive loadings of TPC, TFC, and DPPH on PC1 indicate that this component represented the overall intensity of the polar-functional response, whereas PC2 was associated mainly with variation in the DPPH response. The input matrix used for the time-trajectory PCA is provided in Supplementary Table S12.

TPC and TFC were strongly correlated across the full set of time conditions (r = 0.969), whereas the TPC–DPPH and TFC–DPPH relationships were weaker. After centering the data within each solvent, the corresponding correlations were 0.634 and 0.599, while TPC–TFC remained high (r = 0.929). In our dataset, this indicates that DPPH provided functional information that was partly independent of total phenolic and flavonoid contents. A similar lack of parallelism between polyphenol profiles and antioxidant response has been observed during thyme extraction [29,30]. The temperature trajectory showed a comparable pattern, with PC1 and PC2 together explaining 98.2% of the variance (Figure S2), indicating medium-dependent shifts across the overall TPC–TFC–DPPH profile. The corresponding input matrix is provided in Supplementary Table S13.

#### 2.7.3. Integrated Polar-Functional Response in Relation to the EO Balance

At the solvent-specific reference conditions, IPFI clearly differentiated the extraction media. The highest integrated polar-functional responses were obtained for W and NW, intermediate values for O and G65, and the lowest value for E50. This pattern reflects the combined response of TPC, TFC, and DPPH rather than any single analytical parameter. The source responses, normalized values, and resulting IPFI values are provided in Supplementary Table S10. The sensitivity analysis (Table S15) confirmed this ranking after changing the weights assigned to the three parameters and after excluding NW from the normalization. The only exception occurred when TFC was omitted: for the TPC + DPPH index, G65 (0.458) ranked above O (0.426).

**Figure 5:**
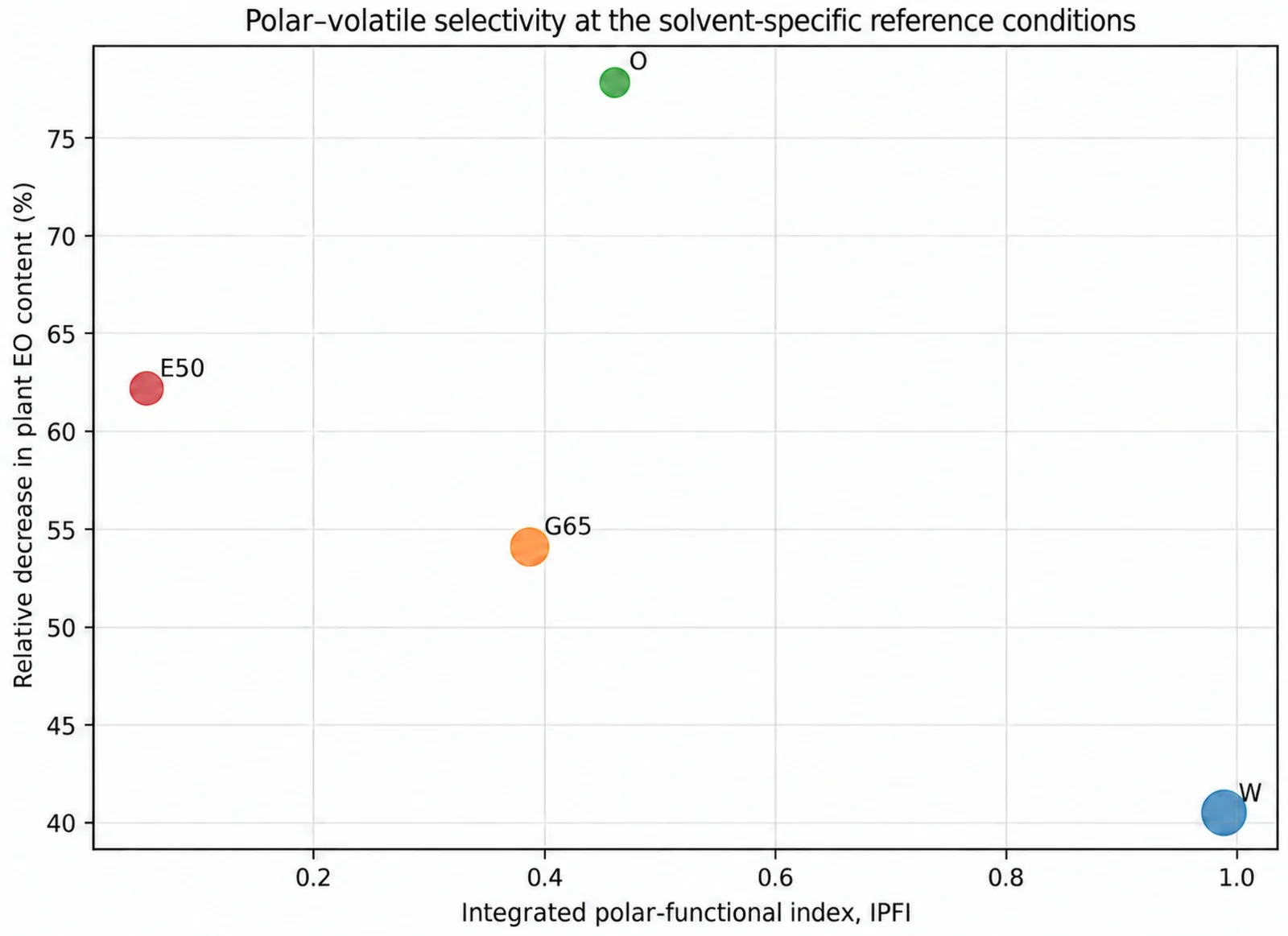
Polar–volatile selectivity map at the solvent-specific reference conditions (W, G65 and O: 100 °C/10 min; E50: 50 °C/10 min). The x-axis shows the integrated polar-functional index (IPFI), the y-axis shows the relative decrease in plant EO content (ΔEO_rel_), and marker size is proportional to thymol AMRI. IPFI normalization included all five solvent systems (W, NW, G65, O and E50), but NW is not shown because a paired EO/GC marker-retention dataset was not available for that system. Solvent codes: W—water; G65—65% glycerol–water; O—rapeseed oil; E50—50% ethanol–water.

Plotting IPFI against the EO balance revealed contrasting fractionation patterns (Figure 5). W combined a high polar-functional response with relatively greater thymol retention, whereas O and E50 were associated with a larger decrease in EO content in the plant material and lower thymol AMRI. G65 occupied an intermediate position and was distinguished by selective retention of γ-terpinene together with relative enrichment of (E)-caryophyllene.

Taken together, the results point to a technological trade-off between the response of the polar fraction and preservation of the oil fraction in the plant material. Water-based systems favored a high TPC–TFC–DPPH response while retaining relatively more thymol, whereas rapeseed oil and 50% ethanol produced a greater decrease in EO content in the biomass. G65 showed an intermediate behavior while also producing characteristic shifts within the volatile-marker profile. From a process-design perspective, the choice of extraction medium should depend on whether the intended outcome is a polar-compound-rich extract, more extensive transfer of the volatile fraction into the extraction phase, or preservation of a particular fingerprint in material intended for subsequent use.

## 3. Materials and Methods

### 3.1. Plant Material

The study was carried out using common thyme herb (*Thymus vulgaris* L.) from a single production batch supplied by Dary Natury Sp. z o.o. (Koryciny, Grodzisk, Poland). The plant material was homogenized by combining representative portions from the same batch, then coarsely ground and passed through a 5.6 mm sieve without further pulverization. The moisture content of the prepared material was measured using a WPS 50 SX moisture analyzer (RADWAG, Radom, Poland) and averaged 9.1%. The material was then stored in plastic containers under conditions limiting exposure to light and moisture. All experimental variants were prepared from the same homogenized batch, thereby reducing the influence of between-batch variability when assessing extraction-induced changes.

### 3.2. Solvents, Reagents, Standards, and Instrumentation

The extraction media were distilled water, plant-derived glycerol (≥99.5%, Ph. Eur.; Galvet, Łambinowice, Poland), analytical-grade ethanol (POCH S.A., Gliwice, Poland), Kujawski rapeseed oil (ZT Kruszwica S.A., Kruszwica, Poland), and commercially available plasma-treated water (nanowater; Nantes Medical, Bolesławiec, Poland). The main marker-retention analysis included data obtained with water, 65% glycerol–water, rapeseed oil, and 50% ethanol–water. The NW medium was the commercial NANTES plasma-treated water used directly from its original packaging. According to the manufacturer’s information recorded in the laboratory documentation at the time of the experiments, the nominal pH of the product was 7.5 and its conductivity was 331 µS/cm; these values were not independently measured in the present study. Nanowater was included only in the complementary comparison of TPC, TFC, and DPPH activity because a complete paired dataset comprising EO content in the post-extraction material and the corresponding GC profile was not available for this system.

Folin–Ciocalteu reagent, sodium carbonate, sodium nitrite (NaNO_2_), aluminum chloride, and sodium hydroxide (analytical grade; POCH S.A., Gliwice, Poland) were used for TPC and TFC determinations. Gallic acid (≥98%) and epicatechin (≥98%) served as calibration standards (Sigma-Aldrich, Buchs, Switzerland). Antioxidant activity was measured using 2,2-diphenyl-1-picrylhydrazyl (DPPH, ≥97%) and Trolox (6-hydroxy-2,5,7,8-tetramethylchromane-2-carboxylic acid, ≥98%; Sigma-Aldrich, Buchs, Switzerland). Xylene and acetone (analytical grade; POCH, Gliwice, Poland), hexane, and anhydrous sodium sulfate (analytical grade; Chempur, Piekary Śląskie, Poland) were used for essential-oil isolation and sample preparation for chromatographic analysis. Dodecane and nonadecane (≥99%; Sigma-Aldrich, Buchs, Switzerland) were used as n-alkane standards for retention-index determination.

Spectrophotometric measurements were performed using a Cary 50 Scan UV–VIS spectrophotometer (Varian, Palo Alto, CA, USA). Extraction was carried out in an IST-3075R orbital shaker (Jeio Tech, Daejeon, Republic of Korea). Essential oils were analyzed using a Varian GC-3800 gas chromatograph equipped with an FID detector (Varian, Walnut Creek, CA, USA) and a Varian GC-3800 system coupled to a Varian 4000 MS/MS mass spectrometer (Varian, Walnut Creek, CA, USA). Additional equipment included analytical balances (RADWAG, Radom, Poland) and an MLL 547 laboratory water bath (AJL Electronic, Kraków, Poland).

### 3.3. Experimental Design and Primary and Secondary Extraction

Conventional solid–liquid extraction was used, based on the general principles of infusion preparation described in PN-ISO 3103, with modifications to the solvent type and extraction temperature [31]. The procedure followed the previously published approach for low-processed extraction of herbal raw materials [16]. A 2.00 g portion of the plant material was placed in a 150 mL beaker and mixed with 100 mL of the appropriate solvent, giving a plant material-to-solvent ratio of 1:50 (*w*/*v*). Initial temperatures of 50, 80, and 100 °C were used for water, aqueous glycerol solutions, rapeseed oil, and nanowater, whereas 50% ethanol was tested at room temperature and at 50 °C. The systems were gently agitated at 130 rpm using an IST-3075R shaker (Jeio Tech, Daejeon, Republic of Korea). Extraction was carried out in 150 mL beakers covered with watch glasses under atmospheric pressure. The stated temperatures refer to the initial temperature of the extraction media at the time of contact with the plant material; no continuous heating or isothermal temperature control was applied during shaking. The resulting extracts were analyzed directly, without subsequent concentration or removal of the extraction media. The reported TPC, TFC, and DPPH values represent analytical concentrations in the liquid extracts as collected after extraction and were determined from the fixed aliquots used in the respective assays. They were not back-calculated to the initial 100 mL solvent charge or converted to total amounts recovered from the plant material. Final extract volume was not measured, and no correction for possible evaporation was applied. Accordingly, the stated 1:50 (*w*/*v*) plant material-to-solvent ratio refers to the initial extraction charge. All experimental variants were prepared in three independent replicates.

In the experiments used to establish the extraction-time range, extraction was carried out for 2–30 min. Comparisons among solvents focused mainly on short extraction times of 2–10 min, whereas the EO marker analysis used paired data obtained after 6 and 10 min at 80 and 100 °C; for 50% ethanol, the corresponding conditions were room temperature and 50 °C. In experiments evaluating the essential-oil balance, the scale was increased tenfold while maintaining the 1:50 (*w*/*v*) ratio, i.e., 20 g of plant material was extracted with 1000 mL of solvent.

After extraction, the liquid phase was separated from the plant material and designated as the primary extract. After separation of the liquid extract, the plant residue was rinsed three times with small portions of distilled water at room temperature; the rinsates were discarded and were not combined with the extract. The purpose of this step was to reduce the amount of freely adhering extraction medium and extract remaining on the surface of the material, rather than to exhaustively remove medium retained within the plant matrix. In the series intended for secondary extraction, the prepared residue was re-extracted with the same medium. In separate series used to determine the residual EO, the entire rinsed residue from the relevant extraction stage was quantitatively transferred to the hydrodistillation flask; the vessels were additionally rinsed with distilled water to ensure quantitative transfer of the material. The resulting extracts and the corresponding plant residues were analyzed in the same manner as those obtained after primary extraction [16]. The coding scheme used for the experimental variants and their role in the subsequent analyses are summarized in Table 3. Rinsing the plant material after separation of the liquid extract should be distinguished from the additional rinsing of the vessels during quantitative transfer of the residue to the hydrodistillation flask. The primary-extraction variant used for residual EO determination ended after the first extraction step, whereas material subjected to secondary extraction underwent an additional contact step with the same medium, as described above.

### 3.4. Determination of Total Phenolic Content (TPC)

Total phenolic content was determined spectrophotometrically using the Folin–Ciocalteu reagent according to a modified Singleton and Rossi method [32], following the procedure described previously [16]. An aliquot of 0.02 mL of the extract and 3.16 mL of distilled water were transferred to a 5 mL volumetric flask. Next, 0.10 mL of Folin–Ciocalteu reagent was added, the mixture was mixed and allowed to stand for 5 min, after which 0.60 mL of saturated Na_2_CO_3_ solution was added. The mixture was mixed again and incubated for 30 min at 40 °C. Absorbance was measured at 765 nm. For TPC determination, a control containing the corresponding extraction medium processed under the same conditions but without plant material was used. The intrinsic absorbance of each extract was measured separately in a system without the color-developing reagent. These control responses were taken into account when calculating the final value, before converting absorbance to gallic acid equivalents. Gallic acid in the range of 10–60 mg/L was used for calibration. Samples were diluted when necessary to keep the response within the linear range of the calibration curve. Results were expressed as milligrams of gallic acid equivalents per liter of extract (mg GAE/L). All determinations were performed in triplicate.

### 3.5. Determination of Total Flavonoid Content (TFC)

Total flavonoid content was determined spectrophotometrically at 510 nm according to the procedure of Karadeniz et al. [33], using the modification described previously [16]. A 1.00 mL aliquot of the extract and 5.00 mL of redistilled water were transferred to a 10 mL volumetric flask, followed by 0.30 mL of 5% (*w*/*w*) aqueous NaNO_2_ solution. After 5 min, 0.60 mL of 10% (*w*/*w*) aqueous AlCl_3_·6H_2_O solution was added. The mixture was mixed, and after a further 5 min, 2.00 mL of 1 M NaOH was introduced. The flask was then filled to volume with redistilled water, and absorbance was measured at 510 nm. For TFC determination, a separate control containing the corresponding extraction medium processed in the same way but without plant material was used, together with a sample of the tested extract prepared without the reagent responsible for color-complex formation. The appropriate corrections were applied before converting absorbance to epicatechin equivalents. Epicatechin in the range of 10–400 mg/L was used for calibration. Results were expressed as milligrams of epicatechin equivalents per liter of extract (mg ECE/L). All determinations were performed in triplicate.

### 3.6. Determination of Antioxidant Activity by the DPPH Assay

Antioxidant activity was determined using the stable DPPH radical according to a modified Brand-Williams et al. method [34]. An alcoholic DPPH solution was prepared and diluted to give an initial absorbance of approximately 0.9 at 517 nm; the solution was protected from light. A 0.020 mL aliquot of the extract was added to 3.00 mL of DPPH solution, mixed thoroughly, and incubated in the dark for 30 min. Absorbance was then measured at 517 nm. In the DPPH assay, the intrinsic absorbance of the extract at 517 nm was corrected using a control prepared without the radical solution. The response of the extract–DPPH system was referenced both to the DPPH control solution and to a similarly prepared control of the extraction medium without plant material. Because antioxidant activity causes a decrease in DPPH absorbance, the response of the solvent itself is not equivalent to a “positive” absorbance of the extract. Antioxidant capacity was expressed as Trolox equivalents using a calibration curve prepared from Trolox solutions in the range of 0.02–2.0 mmol/L. Measurements were performed in triplicate.

### 3.7. Analytical Quality Control of Spectrophotometric Assays

Quality control for all three assays was performed in the five extraction media using repeatability testing and recovery after addition of the appropriate standard to the finished herbal extracts. Ten control determinations were performed for each method–medium combination; gallic acid was added for TPC, epicatechin for TFC, and Trolox for DPPH. The coefficients of variation ranged from 1.02 to 1.43% for TPC, 0.37 to 0.69% for TFC, and 2.88 to 5.15% for DPPH, while recoveries of the respective standards were 96.73–98.92%, 98.83–99.59%, and 89.35–93.89%. All systems met the predefined acceptance criteria of CV < 10% and recovery of 85–115%. The control determinations (*n* = 10) were independent of the three replicates used in the actual extraction experiments.

### 3.8. Determination of Essential-Oil Content in Plant Material

Essential-oil content in the initial plant material and in the material remaining after primary and secondary extraction was determined by hydrodistillation according to the pharmacopoeial procedure [35]. Approximately 20 g of plant material, weighed to the nearest 0.1 g, was placed in a 1000 mL flask and mixed with 400 mL of water. The flask was connected to an essential-oil determination apparatus, cooling was started, and distillation was continued for 3 h from the appearance of the first drop of distillate. After heating was stopped, the apparatus was left for 30 min before the oil volume was read from the graduated section. The oil was transferred to glass vials, dried over anhydrous Na_2_SO_4_, and stored at −20 °C until chromatographic analysis. EO content was expressed as % v/w relative to the mass of plant material. Determinations were performed in triplicate. For post-extraction samples, the entire rinsed residue from the process starting with 20 g of dried commercial plant material was used for the determination. The material was quantitatively transferred to the hydrodistillation flask, with the vessels additionally rinsed with water to ensure complete transfer; no new 20 g portion of the wet residue was weighed. EO content [% *v*/*w*] was expressed relative to the initial mass of the plant material.

The directly measured EO content of the material was used for balance calculations and for the marker-retention indices. For samples in which no distinct oil volume could be read reliably in the graduated receiver, including the secondary E50 variants, xylene was used as a collecting phase for the volatile fraction. In these samples, EO content was recorded as 0.00% *v*/*w* because the oil volume was not volumetrically measurable under the applied procedure. The xylene phase was nevertheless subjected to chromatographic analysis, allowing relative profiling of the recovered volatile constituents. These profiles were used only as relative compositional data and were excluded from quantitative EO-balance and marker-retention calculations.

### 3.9. GC-FID and GC-MS Analysis

Essential oils were analyzed by GC–MS and GC–FID according to the procedure of Kowalski and Wawrzykowski [10], using the parameters applied in the original experiment. Analyses were performed in triplicate. GC–MS was used mainly for compound identification, whereas GC–FID provided the relative proportions of individual constituents in the essential-oil profile.

**GC–MS.** The system consisted of a Varian GC-3800 gas chromatograph coupled to a Varian 4000 MS/MS detector (Varian, Walnut Creek, CA, USA) and fitted with a VF-5MS capillary column (30 m × 0.25 mm × 0.25 µm; stationary phase: 5% phenyl/95% dimethylpolysiloxane). The oven was held at 60 °C for 0.5 min, then heated to 246 °C at 3 °C/min and maintained at 246 °C for 10 min. The injector and transfer-line temperatures were 250 °C. The split ratio was 1:50, and helium was used as the carrier gas at a constant flow rate of 0.5 mL/min. Electron ionization was performed at 70 eV, with mass spectra recorded over the *m*/*z* range 40–1000. The injection volume was 1 µL.

**GC–FID.** Analyses were performed using a Varian GC-3800 gas chromatograph equipped with an FID detector (Varian, Walnut Creek, CA, USA) and a DB-5 column (30 m × 0.25 mm × 0.25 µm; 95% dimethylpolysiloxane/5% phenyl). Chromatographic separation conditions corresponded to those used for GC–MS in the original experiment. The GC–FID signal was used for relative quantification of the essential-oil constituents.

**Qualitative identification.** Essential-oil constituents were identified by comparing experimental retention indices, determined under temperature-programmed conditions according to the method of Van den Dool and Kratz [36], with literature values, including those reported by Adams [37], and by matching the recorded mass spectra with reference spectra in the NIST/EPA/NIH library [38]. The analysis followed the procedure described previously by Kowalski and Wawrzykowski [10].

**Relative quantification.** The relative proportions of identified essential-oil constituents were expressed as area% after normalization of GC–FID peak areas. These profiles were used to compare changes in oil composition among experimental variants and to calculate marker-based indices [39].

### 3.10. Change in Recoverable EO Content

The relative decrease in essential-oil content after extraction was calculated from the directly measured EO content before and after the process:

Δ*EOrel* [%] = [(*Z*_0_ − *Z_pe_*)/*Z*_0_] × 100

where Z_0_ is the EO content of the initial material [% *v*/*w*], and Z_pe_ is the EO content of the corresponding post-extraction material [% *v*/*w*]. The ΔEO_rel_ index describes the relative decrease in the volume of essential oil recoverable from the biomass using the applied procedure, based on the same mass denominator before extraction. It does not measure the mass or volume of essential oil transferred to the liquid extract and should not be interpreted as a complete mass balance of volatile constituents.

### 3.11. Marker Panel and Retention Indices

Six compounds were selected for detailed analysis: myrcene, p-cymene, γ-terpinene, linalool, thymol, and (E)-caryophyllene. The panel included the principal marker of the thymol-type profile, two biosynthetically related compounds, and constituents representing different terpene groups. Selection was based on unambiguous chromatographic identification and the availability of comparable data for the initial material and the post-extraction variants analyzed.

*L_i_,*0 = *EO*_0_ × *C_i_*,0/100

*L_i_,r = EOᵣ* × *C_i_,r*/100

*AMRI_i_* [%] = 100 × *L_i_,r/L_i_*,0

where EO_0_ and EOᵣ are the essential-oil contents of the initial and post-extraction material, respectively [% *v*/*w*]; C_i_,0 and C_i_,r are the relative proportions of marker *i* in the initial and residual essential oil [area%]; and L_i_,0 and L_i_,r are the corresponding operational marker loads of the material. The product EO × area% was used as an operational measure of marker load. AMRI is an apparent retention index derived from the volume of EO recovered using the defined procedure and the relative GC-FID peak area; values above 100% do not indicate an increase in the absolute mass of the compound.

*SEF_i_* = *C_i_,r*/*C_i_,*0

The selective enrichment factor (SEF) describes the change in the relative proportion of a marker in the residual EO. SEF > 1 indicates relative enrichment, SEF = 1 no change in relative proportion, and SEF < 1 relative depletion. An operational thymol-profile balance ratio (CBR) was also calculated:

*CBR* = *thymol*/(*p-cymene* + *γ-terpinene*)

together with the thymol/p-cymene ratio. SEF and CBR were used as descriptors of changes in the relative structure of the marker profile. As derived variables, they were not included again in PCA.

### 3.12. Chemometric Analysis of the Marker Profile

The data matrix X used for marker-profile analysis had dimensions of 29 × 6. Rows represented the initial-material profile, 16 profiles after primary extraction, and 12 profiles after secondary extraction with W, G65, and O; columns represented the six markers. GC–FID proportions are compositional data constrained by a constant sum. For each profile, the six-component subcomposition was therefore reclosed to 100% and then transformed using the centered log-ratio (CLR) transformation according to Aitchison [28]:

*clr*(*x_i_*) = *ln*[*x_i_*/*g*(*x*)]

where g(x) is the geometric mean of the six proportions in a given profile. No zero values were present in the matrix used for PCA, so neither pseudocount replacement nor imputation was required.

**AMRI heatmap.** For primary extraction, a 6 × 16 matrix was constructed, with markers in rows and solvent–temperature–time variants in columns. AMRI values were displayed on their original [%] scale without Z-score standardization, preserving their direct interpretation as the percentage of apparent marker-load retention. Conditions along the x-axis were ordered according to the experimental design (W, G65, O, E50, followed by temperature and time), allowing process-related changes to be followed across the extraction variants.

**PCA of the marker profile.** After CLR transformation, matrix columns were mean-centered without additional autoscaling, and principal component analysis was performed by singular value decomposition (SVD). The PC1–PC2 score plot and marker loadings were used for interpretation. The corresponding PC1 and PC2 marker loadings are reported in Supplementary Table S14. The proportion of variance explained by component *k* was calculated as EV_k_ = s_k_^2^/Σs^2^ × 100, where s_k_ is the corresponding singular value. The PCA included the initial-material profile, 16 profiles after primary extraction, and 12 profiles after secondary extraction with W, G65, and O. Secondary E50 profiles were excluded because residual EO could not be quantified volumetrically. Derived variables (AMRI, SEF, CBR, and EO content) were not used as PCA input variables. The analysis was performed on the mean profiles for each experimental condition; therefore, distances in the PCA score plot were not interpreted as tests of statistical significance between groups.

Marker indices, CLR transformation, PCA, and visualizations were calculated in Python 3.13.5 using NumPy 2.3.5 and Matplotlib 3.10.8. The analyses were exploratory and were used to visualize the similarity structure and the direction of shifts in the marker profile.

### 3.13. Statistical Analysis

Where available, directly measured parameters are presented as mean ± standard deviation (SD) from three independent replicates. Statistical analyses were performed using IBM SPSS Statistics 26. One-way analysis of variance (ANOVA) was applied separately within the individual experimental series, and means were compared using Tukey’s test at *p* < 0.05. Means that did not share a common letter were considered significantly different at *p* < 0.05.

AMRI, SEF, CBR, log_2_ fold change, and IPFI, as well as the PCA analyses, were calculated from condition means and treated as descriptive and exploratory analyses. No additional inferential tests were applied to these derived quantities.

### 3.14. Multiresponse Analysis of TPC, TFC, and DPPH

The effects of time and temperature were examined as two separate experimental trajectories: changes in extraction time at a fixed initial temperature, and changes in initial temperature at a fixed extraction time of 10 min.

**Process sensitivity matrix.** A dimensionless logarithmic fold change was used to compare the magnitude of the responses to time and temperature. For each response Y (TPC, TFC, or DPPH), the time effect was calculated as:

S_time_ = log_2_(Y_10_ min/Y_2_ min)

and the temperature effect as:

S_temperature_ = log_2_(Y_100_ °C/Y_50_ °C)

with both temperature points referring to 10 min extraction. For E50, only the time component was analyzed (2–10 min at 50 °C) because this system followed a different temperature scheme. A value of S = 1 indicates a twofold increase in the response, S = 0 no change, and S < 0 a decrease. The S values were visualized as a heatmap, with solvent–process factor combinations in rows and TPC, TFC, and DPPH in columns.

**Integrated polar-functional response.** To condense the three parallel responses into a single descriptive index, each variable was normalized to the maximum value obtained among the systems being compared:

n_j_ = Y_j_/max(Y_j_)

The integrated polar-functional index (IPFI) was then calculated as the geometric mean of the normalized TPC, TFC, and DPPH values:

IPFI = (n_TPC_ × n_TFC_ × n_DPPH_)^1/3^

Equal weights were assigned to TPC, TFC, and DPPH. IPFI was used as a composite measure of the integrated polar-functional response of the extraction systems. In the polar–volatile selectivity map, IPFI was plotted on the x-axis, the relative decrease in EO content in the plant material (ΔEO_rel_) on the y-axis, and marker size was proportional to thymol AMRI. IPFI sensitivity was evaluated by: (i) re-normalizing the data after excluding NW, (ii) doubling the weight of one response at a time (2:1:1, 1:2:1, and 1:1:2), and (iii) calculating geometric means for each pair of responses. In the primary analysis, normalization included all five media, and the detailed results are presented in Table S15.

**PCA and correlations for TPC–TFC–DPPH.** Two separate exploratory PCAs were performed after autoscaling each variable to a mean of 0 and a standard deviation of 1, which accounted for the different units and numerical ranges of TPC, TFC, and DPPH. The time-trajectory PCA included five systems (W, NW, G65, O, and E50) at four extraction times of 2, 4, 6, and 10 min, giving a 20 × 3 matrix. The temperature-trajectory PCA included W, NW, G65, and O at 50, 80, and 100 °C after 10 min of extraction, giving a 12 × 3 matrix. E50 was not included in the temperature PCA. PCA was performed by SVD on the autoscaled matrix, corresponding to an analysis based on the correlation matrix; interpretation was based on PC1–PC2 scores, variable loadings, and the percentage of explained variance. IPFI was not included as an input variable because it is mathematically derived from TPC, TFC, and DPPH. Pearson correlations among TPC, TFC, and DPPH were calculated from condition means. Correlations were also calculated after centering the results within each solvent to reduce the dominant effect of differences among extraction media. These correlations were descriptive and were not used for hypothesis testing at the replicate level.

## 4. Conclusions

Under the conditions tested, short-term extraction of thyme herb altered both the amount of essential oil recoverable from the residual biomass and the relative proportions of the six GC-FID markers. The highest apparent thymol retention was observed after water extraction (AMRI 59.2%), whereas rapeseed oil and 50% ethanol were associated with a greater decrease in recoverable EO. Glycerol favored relative enrichment of the profile in selected constituents, particularly (E)-caryophyllene, while the relative proportion of linalool was more pronounced after ethanol extraction.

Water and nanowater produced the highest integrated TPC/TFC/DPPH response, although NW was not included in the analysis of EO marker retention. Combining residual EO measurement with the relative chromatographic profile, exploratory AMRI/SEF indices, and CLR-PCA provides useful criteria for process selection and for assessing the potential for sequential use of the remaining biomass.

The conclusions are limited to a single batch of plant material and to comparisons of medium-specific reference conditions, with a different initial temperature for ethanol. The indices are operational and should not be interpreted as a balance of the absolute amounts of individual markers or as evidence of a change in the biologically determined chemotype.

## 5. Limitations

The study has several limitations that should be considered when interpreting the results. All experiments were carried out using a single homogenized commercial batch of *T. vulgaris* and one particle-size fraction. The findings therefore apply specifically to this thymol-type material under the extraction conditions tested and cannot be directly generalized to other chemotypes, cultivars, harvest stages, or production batches.

AMRI and the related marker indices combine the directly measured residual EO content with relative GC-FID area%. They are operational indices rather than absolute measures of individual compounds. AMRI values above 100% reflect relative enrichment of a marker within the residual EO fraction and should not be interpreted as an increase in its absolute amount. Similarly, ΔEO_rel_ describes the decrease in EO content of the solid plant material but cannot distinguish between transfer of volatile compounds into the extraction phase and losses that may occur during processing.

The marker-profile PCA was based on six selected constituents and does not capture the full 46-compound essential-oil profile. Time and temperature were evaluated in separate experimental series rather than in a full factorial design, so their interaction could not be estimated. Because the extraction vessels were covered with watch glasses but were not sealed, some evaporation, particularly in variants started at 100 °C, cannot be excluded. Final extract volume and the liquid temperature during and at the end of extraction were not measured. Consequently, the reported TPC, TFC, and DPPH values were not corrected for possible evaporation-related concentration effects, and comparisons among temperature conditions should therefore be interpreted as responses of the defined technological protocols rather than as evaporation-corrected extraction yields. Nanowater was included in the TPC/TFC/DPPH comparison, but not in the EO marker-retention analysis, because a complete paired dataset linking residual EO content with the corresponding GC profile was not available for this system. TPC, TFC, and DPPH describe the overall analytical response of the extracts, which depends on the composition and properties of the extraction medium, rather than the concentrations of individual metabolites.

Future work could extend this approach to additional thyme batches and chemotypes and include absolute quantification of selected volatile markers and individual phenolic compounds. Validation under larger-scale processing conditions would also help assess the broader technological applicability of the proposed approach.

## Figures and Tables

**Table 1:** Main constituents of the initial essential oil included in the marker analysis.

Constituent	Relative Abundance [%]	Role in Analysis
myrcene	0.66	monoterpene marker
p-cymene	21.53	thymol-type profile constituent/γ-terpinene–p-cymene–thymol system
γ-terpinene	5.28	biosynthetically related to the thymol-type profile
linalool	1.90	oxygenated monoterpene marker/alternative profile marker
thymol	53.65	principal marker of the thymol-type profile
(E)-caryophyllene	1.50	sesquiterpene marker

**Table 2:** Selected AMRI and SEF values after primary extraction at the solvent-specific reference conditions (W, G65 and O: 100 °C/10 min; E50: 50 °C/10 min).

System	Marker	GC–FID Area in Residual EO [%]	Residual EO [% *v*/*w*]	AMRI [%]	SEF
Water 100 °C	myrcene	0.69	1.10	62.2	1.05
Water 100 °C	p-cymene	15.40		42.5	0.72
Water 100 °C	γ-terpinene	6.18		69.6	1.17
Water 100 °C	linalool	1.56		48.8	0.82
Water 100 °C	thymol	53.40		59.2	1.00
Water 100 °C	(E)-caryophyllene	1.80		71.4	1.20
Glycerol 65% 100 °C	myrcene	0.69	0.85	48.0	1.05
Glycerol 65% 100 °C	p-cymene	9.42		20.1	0.44
Glycerol 65% 100 °C	γ-terpinene	8.75		76.1	1.66
Glycerol 65% 100 °C	linalool	1.39		33.6	0.73
Glycerol 65% 100 °C	thymol	45.92		39.3	0.86
Glycerol 65% 100 °C	(E)-caryophyllene	4.89		149.8	3.26
Rapeseed oil 100 °C	myrcene	0.73	0.41	24.5	1.11
Rapeseed oil 100 °C	p-cymene	13.98		14.4	0.65
Rapeseed oil 100 °C	γ-terpinene	10.14		42.6	1.92
Rapeseed oil 100 °C	linalool	2.89		33.7	1.52
Rapeseed oil 100 °C	thymol	44.59		18.4	0.83
Rapeseed oil 100 °C	(E)-caryophyllene	4.79		70.8	3.19
Ethanol 50% 50 °C	myrcene	0.09	0.70	5.2	0.14
Ethanol 50% 50 °C	p-cymene	9.12		16.0	0.42
Ethanol 50% 50 °C	γ-terpinene	5.60		40.1	1.06
Ethanol 50% 50 °C	linalool	4.12		82.0	2.17
Ethanol 50% 50 °C	thymol	38.60		27.2	0.72
Ethanol 50% 50 °C	(E)-caryophyllene	3.12		78.7	2.08

AMRI—apparent marker retention index; SEF—selective enrichment factor. AMRI combines residual EO content with the relative GC-FID contribution of a marker and expresses the apparent residual marker load relative to the initial material (100%). SEF describes the change in the relative proportion of a marker in the residual EO; SEF > 1 indicates relative enrichment, whereas SEF < 1 indicates relative depletion. AMRI and SEF are derived descriptive indices. EO content [% *v*/*w*] was expressed relative to the initial mass of the dried plant material; AMRI and SEF were calculated from the mean values for each experimental condition. Direct measurements were performed in triplicate (*n* = 3), whereas no separate SD was calculated for the derived values.

**Table 3:** Coding scheme for the experimental variants used in the study.

System	Code	Temperature in Marker Analysis	Extraction Time [min]	Example Code	Use in Analysis
Distilled water	W	80 and 100 °C	6 and 10	P-W-100-10/S-W-100-10	EO, GC, AMRI/SEF/CBR; also TPC/TFC/DPPH
65% glycerol–water	G65	80 and 100 °C	6 and 10	P-G65-100-10/S-G65-100-10	EO, GC, AMRI/SEF/CBR; also TPC/TFC/DPPH
Rapeseed oil	O	80 and 100 °C	6 and 10	P-O-100-10/S-O-100-10	EO, GC, AMRI/SEF/CBR; also TPC/TFC/DPPH
50% ethanol–water	E50	room temperature and 50 °C	6 and 10	P-E50-50-10/S-E50-50-10	EO and GC; secondary E50 excluded from new indices when EO = 0.00% *v*/*w*
Nanowater	NW	50, 80 and 100 °C *	2–10 *	P-NW-100-10	TPC/TFC/DPPH context only; no marker-retention analysis

P—primary extraction; S—secondary extraction. The code format is stage–solvent–temperature–time. * During preliminary time-range testing, extraction was extended up to 30 min.

## Data Availability

The data supporting the findings of this study are contained within the article and its Supplementary Materials.

## Supplementary Materials

The following supporting information can be downloaded at: https://www.mdpi.com/article/10.3390/molecules31213796/s1, The Supplementary Materials include Tables S1–S15, containing condition-level source data, derived indices, PCA matrices, CLR-PCA loadings, and the IPFI sensitivity analysis (S15), as well as Figures S1 and S2, showing the exploratory PCA trajectories for extraction time and temperature.

## Abbreviations

**The following abbreviations are used in this manuscript**: AMRI, apparent marker retention index; ANOVA, analysis of variance; CBR, thymol-profile balance ratio; CLR, centered log-ratio; DPPH, 2,2-diphenyl-1-picrylhydrazyl; E50, 50% ethanol–water; ECE, epicatechin equivalents; EI, electron ionization; EMA, European Medicines Agency; EO, essential oil; G65, 65% glycerol–water; GAE, gallic acid equivalents; GC-FID, gas chromatography–flame ionization detection; GC-MS, gas chromatography–mass spectrometry; IPFI, integrated polar-functional index; NW, nanowater; O, rapeseed oil; PCA, principal component analysis; RT, room temperature; SD, standard deviation; SEF, selective enrichment factor; SVD, singular value decomposition; TE, Trolox equivalents; TFC, total flavonoid content; TPC, total phenolic content; UV–VIS, ultraviolet–visible; W, water; ΔEO_rel_, relative decrease in essential-oil content.
