# Supplementary material for: Solvent-Dependent Selective Retention of Essential-Oil Markers and Modification of the Thymol-Type Chemical Fingerprint in Post-Extraction Thymus vulgaris L. Biomass
Source: Molecules. 2026 Oct 22;31(21):3796. doi: 10.3390/molecules31213796 (PMC13764722; doi:10.3390/molecules31213796)
Supplement: Supplementary file 1 [file molecules-31-03796-s001.zip › molecules-4622388-supplementary.pdf]

Supplementary Materials – Source Data and Derived Indices for Thymus vulgaris Extraction Study

Data note. Tables S1–S4 report mean ± SD values and Tukey homogeneous-group letters from the original experimental series. Tables S5–S7 report condition means and the original Tukey homogeneous-group letters from the original experimental series. Derived indices and PCA input matrices were calculated from the corresponding condition means. Solvent codes used throughout: W—water; NW—nanowater; G65—65% glycerol–water; O—rapeseed oil; E50—50% ethanol–water; RT—room temperature.

Table S1. Total phenolic content (TPC) and total flavonoid content (TFC) during primary extraction as a function of extraction time.

Code	Solvent	Temp. (°C)	Time (min)	TPC (mg GAE/L)	TFC (mg ECE/L)
W	Water	100	2	397.83 ± 13.15^{b}	249.57 ± 13.99^{c}
W	Water	100	4	453.91 ± 19.07^{b}	329.18 ± 10.10^{b}
W	Water	100	6	804.48 ± 55.75^{a}	503.18 ± 21.64^{a}
W	Water	100	10	860.69 ± 75.95^{a}	518.57 ± 38.87^{a}
NW	Nanowater	100	2	222.17 ± 12.95^{d}	180.26 ± 15.47^{c}
NW	Nanowater	100	4	310.45 ± 17.60^{c}	194.12 ± 8.36^{c}
NW	Nanowater	100	6	539.60 ± 27.83^{b}	289.87 ± 19.64^{b}
NW	Nanowater	100	10	573.49 ± 17.30^{a}	412.05 ± 29.30^{a}
G65	65% glycerol-water	100	2	207.25 ± 16.15^{d}	74.12 ± 4.30^{c}
G65	65% glycerol-water	100	4	219.25 ± 19.52^{c}	99.85 ± 7.66^{b}
G65	65% glycerol-water	100	6	257.46 ± 21.06^{b}	137.97 ± 10.14^{a}
G65	65% glycerol-water	100	10	395.16 ± 19.93^{a}	143.60 ± 11.84^{a}
O	Rapeseed oil	100	2	294.53 ± 13.55^{d}	179.69 ± 8.18^{c}
O	Rapeseed oil	100	4	323.29 ± 16.84^{c}	182.75 ± 14.04^{bc}
O	Rapeseed oil	100	6	391.21 ± 14.19^{b}	191.85 ± 13.37^{b}
O	Rapeseed oil	100	10	409.24 ± 24.79^{a}	279.91 ± 16.69^{a}
E50	50% ethanol-water	50	2	14.75 ± 0.58^{d}	7.15 ± 0.62^{c}
E50	50% ethanol-water	50	4	24.50 ± 1.38^{c}	7.12 ± 0.50^{c}
E50	50% ethanol-water	50	6	38.20 ± 1.74^{b}	23.20 ± 0.71^{b}
E50	50% ethanol-water	50	10	47.12 ± 3.81^{a}	35.20 ± 2.22^{a}

Values are presented as mean ± SD (n = 3). Within each solvent and response variable (TPC or TFC), means that do not share a superscript letter differ significantly according to Tukey's HSD test ($p < 0.05$).

Table S2. TPC and TFC during primary extraction as a function of extraction temperature (10 min).

Code	Solvent	Temp. (°C)	Time (min)	TPC (mg GAE/L)	TFC (mg ECE/L)
W	Water	50	10	555.78 ± 21.78^{c}	308.15 ± 14.92^{c}
W	Water	80		736.33 ± 41.27^{b}	462.40 ± 32.33^{b}
W	Water	100		860.69 ± 75.95^{a}	518.57 ± 38.87^{a}
NW	Nanowater	50		336.49 ± 14.10^{c}	134.66 ± 6.52^{c}
NW	Nanowater	80		394.80 ± 19.97^{b}	219.90 ± 12.85^{b}
NW	Nanowater	100		573.49 ± 17.30^{a}	412.05 ± 29.30^{a}
G65	65% glycerol-water	50		206.37 ± 9.16^{c}	144.25 ± 11.66^{ab}
G65	65% glycerol-water	80		293.51 ± 23.80^{b}	147.65 ± 9.48^{a}
G65	65% glycerol-water	100		395.16 ± 19.93^{a}	143.60 ± 11.84^{b}
O	Rapeseed oil	50		287.20 ± 25.62^{c}	160.02 ± 7.67^{b}
O	Rapeseed oil	80		397.50 ± 24.32^{b}	280.20 ± 18.79^{a}
O	Rapeseed oil	100		409.24 ± 24.79^{a}	279.91 ± 16.69^{a}

Values are presented as mean ± SD (n = 3). Within each solvent and response variable (TPC or TFC), means that do not share a superscript letter differ significantly according to Tukey's HSD test ($p < 0.05$).

Table S3. DPPH antioxidant response during primary extraction as a function of extraction time.

Code	Solvent	Temp. (°C)	Time (min)	DPPH (mM TE)
W	Water	100	2	1.0350^{c} ± 0.0373
W	Water	100	4	1.1440^{b} ± 0.0380
W	Water	100	6	1.1760^{ab} ± 0.0729
W	Water	100	10	1.1990^{a} ± 0.0366
NW	Nanowater	100	2	1.0070^{d} ± 0.0413
NW	Nanowater	100	4	1.1120^{c} ± 0.0434
NW	Nanowater	100	6	1.1800^{b} ± 0.0649
NW	Nanowater	100	10	1.2400^{a} ± 0.0459
G65	65% glycerol-water	100	2	0.1021^{d} ± 0.0051
G65	65% glycerol-water	100	4	0.1502^{c} ± 0.0131
G65	65% glycerol-water	100	6	0.3649^{b} ± 0.0282
G65	65% glycerol-water	100	10	0.5672^{a} ± 0.0413
O	Rapeseed oil	100	2	0.1340^{d} ± 0.0075
O	Rapeseed oil	100	4	0.2540^{c} ± 0.0225
O	Rapeseed oil	100	6	0.3560^{b} ± 0.0305
O	Rapeseed oil	100	10	0.4735^{a} ± 0.0404
E50	50% ethanol-water	50	2	0.0164^{d} ± 0.0013
E50	50% ethanol-water	50	4	0.0247^{c} ± 0.0013
E50	50% ethanol-water	50	6	0.0431^{b} ± 0.0033
E50	50% ethanol-water	50	10	0.0541^{a} ± 0.0019

DPPH values are presented as mean ± SD (n = 3). Within each solvent, means that do not share a superscript letter differ significantly according to Tukey's HSD test ($p < 0.05$).

Table S4. DPPH antioxidant response during primary extraction as a function of extraction temperature (10 min).

Code	Solvent	Temp. (°C)	Time (min)	DPPH (mM TE)
W	Water	50	10	0.5623^{c} ± 0.0376
W	Water	80		0.9834^{b} ± 0.0824
W	Water	100		1.1990^{a} ± 0.0366
NW	Nanowater	50		0.6120^{c} ± 0.0373
NW	Nanowater	80		0.9652^{b} ± 0.0734
NW	Nanowater	100		1.2400^{a} ± 0.0459
G65	65% glycerol-water	50		0.2134^{c} ± 0.0115
G65	65% glycerol-water	80		0.3892^{b} ± 0.0288
G65	65% glycerol-water	100		0.5672^{a} ± 0.0413
O	Rapeseed oil	50		0.2443^{c} ± 0.0186
O	Rapeseed oil	80		0.3214^{b} ± 0.0225
O	Rapeseed oil	100		0.4735^{a} ± 0.0404

E50 was not included in the temperature-trajectory PCA because its experimental temperature scheme was not directly comparable. DPPH values are presented as mean ± SD (n = 3). Within each solvent, means that do not share a superscript letter differ significantly according to Tukey's HSD test ($p < 0.05$).

Table S5. Residual essential-oil (EO) content and relative EO balance after primary and secondary extraction.

Code	Temperature (°C)	Time (min)	EO after primary (% v/w)	EO remaining primary (%)	ΔEO_{rel} primary (%)	EO after secondary (% v/w)	EO remaining secondary (%)	ΔEO_{rel} secondary (%)
W	100	10	1.10^{cd}	59.46	40.54	0.60^{e}	32.43	67.57
W	100	6	1.20^{c}	64.86	35.14	0.65^{d}	35.14	64.86
W	80	10	1.26^{b}	68.11	31.89	0.75^{c}	40.54	59.46
W	80	6	1.30^{b}	70.27	29.73	0.80^{b}	43.24	56.76
G65	100	10	0.85^{d}	45.95	54.05	0.65^{d}	35.14	64.86
G65	100	6	0.90^{d}	48.65	51.35	0.75^{c}	40.54	59.46
G65	80	10	1.25^{b}	67.57	32.43	0.55^{f}	29.73	70.27
G65	80	6	1.35^{b}	72.97	27.03	0.60^{e}	32.43	67.57
O	100	10	0.41^{i}	22.16	77.84	0.05^{g}	2.70	97.30
O	100	6	0.50^{h}	27.03	72.97	0.04^{g}	2.16	97.84
O	80	10	0.55^{g}	29.73	70.27	0.01^{i}	0.54	99.46
O	80	6	0.55^{g}	29.73	70.27	0.03^{h}	1.62	98.38
E50	RT	10	0.75^{e}	40.54	59.46	0.00^{i}	0.00	100.00
E50	RT	6	0.85^{d}	45.95	54.05	0.00^{i}	0.00	100.00
E50	50	10	0.70^{f}	37.84	62.16	0.00^{i}	0.00	100.00
E50	50	6	0.75^{e}	40.54	59.46	0.00^{i}	0.00	100.00

Initial EO content was 1.85% v/w (Tukey group a). Within each extraction stage (primary or secondary), EO means that do not share a superscript letter differ significantly according to Tukey's HSD test ($p < 0.05$).

For secondary E50 variants, 0.00% v/w denotes the absence of a volumetrically measurable EO layer under the applied hydrodistillation procedure and should not be interpreted as the complete absence of chromatographically detectable volatile constituents.

Table S6. Relative GC-FID area (%) of the six-marker panel in the initial material and after primary extraction.

Code	Temperature (°C)	Time (min)	Myrcene	p-Cymene	γ-Terpinene	Linalool	Thymol	(E)-Caryophyllene
Initial	-	-	0.66^{g}	21.53^{o}	5.28^{o}	1.90^{c}	53.65^{q}	1.50^{a}
W	100	10	0.69^{f}	15.40^{a}	6.18^{k}	1.56^{j}	53.40^{b}	1.80^{j}
W	100	6	0.71^{e}	13.20^{e}	5.75^{l}	1.76^{i}	52.10^{c}	1.90^{ij}
W	80	10	0.75^{c}	12.75^{g}	7.52^{j}	1.97^{g}	54.98^{a}	1.96^{hi}
W	80	6	0.03^{m}	14.75^{c}	9.11^{c}	1.60^{j}	49.27^{d}	3.24^{e}
G65	100	10	0.69^{f}	9.42^{l}	8.75^{d}	1.39^{k}	45.92^{h}	4.89^{b}
G65	100	6	0.71^{e}	8.65^{n}	7.89^{g}	1.45^{k}	47.20^{f}	3.15^{e}
G65	80	10	1.03^{a}	15.01^{b}	8.16^{f}	1.89^{h}	48.65^{e}	2.61^{f}
G65	80	6	0.89^{b}	12.56^{h}	7.80^{h}	1.56^{j}	46.75^{g}	2.45^{g}
O	100	10	0.73^{d}	13.98^{d}	10.14^{a}	2.89^{e}	44.59^{i}	4.79^{b}
O	100	6	0.60^{h}	12.80^{f}	9.85^{b}	1.89^{h}	41.20^{j}	3.80^{d}
O	80	10	0.61^{h}	11.50^{j}	8.65^{e}	2.12^{f}	39.80^{l}	4.12^{c}
O	80	6	0.52^{i}	12.30^{i}	7.58^{i}	1.85^{h}	40.50^{k}	3.85^{d}
E50	RT	10	0.05^{l}	9.15^{m}	5.60^{m}	2.12^{f}	35.20^{p}	3.12^{e}
E50	RT	6	0.07^{k}	8.65^{n}	4.86^{n}	3.02^{d}	37.40^{n}	2.05^{h}
E50	50	10	0.09^{j}	9.12^{m}	5.60^{m}	4.12^{a}	38.60^{m}	3.12^{e}
E50	50	6	0.08^{jk}	10.20^{k}	4.85^{n}	3.89^{b}	37.20^{o}	4.02^{c}

Values are relative GC-FID area%. Within each constituent column, values that do not share a superscript letter differ significantly according to Tukey's HSD test ($p < 0.05$).

Table S7. Relative GC-FID area (%) of the six-marker panel in the initial material and after secondary extraction.

Code	Temperature (°C)	Time (min)	Myrcene	p-Cymene	γ-Terpinene	Linalool	Thymol	(E)-Caryophyllene
Initial	-	-	0.66^{g}	21.53^{a}	5.28^{g}	1.90^{a}	53.65^{a}	1.50^{a}
W	100	10	0.45^{h}	13.20^{h}	4.12^{f}	1.02^{h}	40.50^{i}	1.40^{ij}
W	100	6	0.41^{i}	12.90^{i}	4.16^{f}	1.32^{g}	39.80^{j}	1.30^{j}
W	80	10	0.21^{j}	10.20^{j}	4.16^{f}	1.02^{h}	38.60^{k}	1.45^{i}
W	80	6	0.92^{b}	15.74^{g}	5.70^{e}	1.80^{ef}	39.80^{j}	2.71^{e}
G65	100	10	0.91^{b}	17.40^{f}	5.70^{e}	1.74^{f}	46.50^{c}	2.14^{g}
G65	100	6	0.95^{a}	18.20^{d}	6.12^{c}	1.83^{de}	45.20^{d}	0.26^{k}
G65	80	10	0.89^{c}	18.60^{b}	5.85^{d}	1.84^{de}	47.10^{b}	1.89^{h}
G65	80	6	0.92^{b}	18.50^{c}	6.10^{c}	1.89^{d}	47.26^{a}	2.46^{f}
O	100	10	0.75^{f}	17.60^{e}	5.86^{d}	2.15^{c}	42.80^{g}	2.85^{d}
O	100	6	0.78^{e}	19.02^{a}	6.12^{c}	2.13^{c}	42.60^{h}	3.14^{c}
O	80	10	0.74^{f}	18.52^{c}	6.32^{b}	1.90^{d}	43.60^{e}	3.19^{c}
O	80	6	0.83^{d}	19.01^{a}	6.38^{a}	2.96^{b}	43.06^{f}	3.74^{b}
E50	RT	10	0.02^{kl}	4.65^{n}	2.15^{h}	0.50^{i}	29.40^{o}	2.85^{d}
E50	RT	6	0.01^{l}	5.12^{l}	1.85^{j}	0.46^{ij}	32.50^{l}	1.85^{h}
E50	50	10	0.03^{k}	4.70^{m}	2.02^{i}	0.38^{k}	31.56^{m}	1.96^{h}
E50	50	6	0.02^{kl}	6.02^{k}	1.75^{k}	0.41^{jk}	29.80^{n}	2.12^{g}

Values are relative GC-FID area%. Within each constituent column, values that do not share a superscript letter differ significantly according to Tukey's HSD test ($p < 0.05$). Secondary E50 values represent relative GC-FID profiles of volatile constituents detected in the xylene collection phase despite the absence of a volumetrically measurable EO layer. These profiles were therefore excluded from AMRI calculations and CLR-PCA.

Table S8. Source values and derived AMRI/SEF indices at the solvent-specific reference conditions.

Stage	Code	Marker	Residual EO (% v/w)	Residual marker area (%)	Initial operational marker load	Residual operational marker load	AMRI (%)	SEF
Primary	W	myrcene	1.10	0.69[f]	0.01221	0.00759	62.2	1.045
Primary	W	p-cymene	1.10	15.40[a]	0.39831	0.16940	42.5	0.715
Primary	W	γ-terpinene	1.10	6.18[k]	0.09768	0.06798	69.6	1.170
Primary	W	linalool	1.10	1.56[j]	0.03515	0.01716	48.8	0.821
Primary	W	thymol	1.10	53.40[b]	0.99252	0.58740	59.2	0.995
Primary	W	(E)-caryophyllene	1.10	1.80[j]	0.02775	0.01980	71.4	1.200
Primary	G65	myrcene	0.85	0.69[f]	0.01221	0.00586	48.0	1.045
Primary	G65	p-cymene	0.85	9.42[l]	0.39831	0.08007	20.1	0.438
Primary	G65	γ-terpinene	0.85	8.75[d]	0.09768	0.07437	76.1	1.657
Primary	G65	linalool	0.85	1.39[k]	0.03515	0.01181	33.6	0.732
Primary	G65	thymol	0.85	45.92[h]	0.99252	0.39032	39.3	0.856
Primary	G65	(E)-caryophyllene	0.85	4.89[b]	0.02775	0.04156	149.8	3.260
Primary	O	myrcene	0.41	0.73[d]	0.01221	0.00299	24.5	1.106
Primary	O	p-cymene	0.41	13.98[d]	0.39831	0.05732	14.4	0.649
Primary	O	γ-terpinene	0.41	10.14[a]	0.09768	0.04157	42.6	1.920
Primary	O	linalool	0.41	2.89[e]	0.03515	0.01185	33.7	1.521
Primary	O	thymol	0.41	44.59[i]	0.99252	0.18282	18.4	0.831
Primary	O	(E)-caryophyllene	0.41	4.79[b]	0.02775	0.01964	70.8	3.193
Primary	E50	myrcene	0.70	0.09[j]	0.01221	0.00063	5.2	0.136
Primary	E50	p-cymene	0.70	9.12[m]	0.39831	0.06384	16.0	0.424
Primary	E50	γ-terpinene	0.70	5.60[m]	0.09768	0.03920	40.1	1.061
Primary	E50	linalool	0.70	4.12[a]	0.03515	0.02884	82.0	2.168
Primary	E50	thymol	0.70	38.60[m]	0.99252	0.27020	27.2	0.719
Primary	E50	(E)-caryophyllene	0.70	3.12[e]	0.02775	0.02184	78.7	2.080
Secondary	W	myrcene	0.60	0.45[h]	0.01221	0.00270	22.1	0.682
Secondary	W	p-cymene	0.60	13.20[h]	0.39831	0.07920	19.9	0.613
Secondary	W	γ-terpinene	0.60	4.12[f]	0.09768	0.02472	25.3	0.780
Secondary	W	linalool	0.60	1.02[h]	0.03515	0.00612	17.4	0.537
Secondary	W	thymol	0.60	40.50[i]	0.99252	0.24300	24.5	0.755
Secondary	W	(E)-caryophyllene	0.60	1.40[ij]	0.02775	0.00840	30.3	0.933
Secondary	G65	myrcene	0.65	0.91[b]	0.01221	0.00592	48.4	1.379
Secondary	G65	p-cymene	0.65	17.40[f]	0.39831	0.11310	28.4	0.808
Secondary	G65	γ-terpinene	0.65	5.70[e]	0.09768	0.03705	37.9	1.080
Secondary	G65	linalool	0.65	1.74[f]	0.03515	0.01131	32.2	0.916
Secondary	G65	thymol	0.65	46.50[c]	0.99252	0.30225	30.5	0.867
Secondary	G65	(E)-caryophyllene	0.65	2.14[g]	0.02775	0.01391	50.1	1.427
Secondary	O	myrcene	0.05	0.75[f]	0.01221	0.00038	3.1	1.136
Secondary	O	p-cymene	0.05	17.60[e]	0.39831	0.00880	2.2	0.817
Secondary	O	γ-terpinene	0.05	5.86[d]	0.09768	0.00293	3.0	1.110
Secondary	O	linalool	0.05	2.15[c]	0.03515	0.00108	3.1	1.132
Secondary	O	thymol	0.05	42.80[g]	0.99252	0.02140	2.2	0.798
Secondary	O	(E)-caryophyllene	0.05	2.85[d]	0.02775	0.00143	5.1	1.900

Superscript letters attached to residual marker area values denote Tukey homogeneous groups within the corresponding constituent and extraction stage (primary or secondary) ($p < 0.05$). Letters should not be compared between primary and secondary extraction. AMRI and SEF are derived descriptive indices and were not subjected to additional post-hoc testing. Secondary E50 was not calculated because residual EO could not be quantified volumetrically.

Table S9. Thymol-profile balance ratios at the solvent-specific reference conditions.

Stage	Code	Temperature (°C)	Time (min)	Thymol (%)	p-Cymene (%)	γ-Terpinene (%)	Thymol/p-Cymene	CBR
Primary	W	100	10	53.40	15.40	6.18	3.468	2.475
Primary	G65	100	10	45.92	9.42	8.75	4.875	2.527
Primary	O	100	10	44.59	13.98	10.14	3.190	1.849
Primary	E50	50	10	38.60	9.12	5.60	4.232	2.622
Secondary	W	100	10	40.50	13.20	4.12	3.068	2.338
Secondary	G65	100	10	46.50	17.40	5.70	2.672	2.013
Secondary	O	100	10	42.80	17.60	5.86	2.432	1.824

CBR = thymol/(p-cymene + γ-terpinene).

Table S10. Source responses and integrated polar-functional index (IPFI) at the solvent-specific reference conditions.

Code	Solvent	Temperature (°C)	TPC (mg GAE/L)	TFC (mg ECE/L)	DPPH (mM TE)	nTPC	nTFC	nDPPH	IPFI
W	Water	100	860.69	518.57	1.1990	1.000	1.000	0.967	0.989
NW	Nanowater	100	573.49	412.05	1.2400	0.666	0.795	1.000	0.809
G65	65% glycerol-water	100	395.16	143.60	0.5672	0.459	0.277	0.457	0.387
O	Rapeseed oil	100	409.24	279.91	0.4735	0.475	0.540	0.382	0.461
E50	50% ethanol-water	50	47.12	35.20	0.0541	0.055	0.068	0.044	0.055

IPFI = (nTPC × nTFC × nDPPH)^(1/3), where each response was normalized to the maximum value observed among the compared solvent systems.

Table S11. Process-sensitivity indices used in the Process Sensitivity Matrix.

Contrast	Code	$\log_2$ FC TPC	$\log_2$ FC TFC	$\log_2$ FC DPPH
Time 2→10 min	W	1.113	1.055	0.212
Time 2→10 min	NW	1.368	1.193	0.300
Time 2→10 min	G65	0.931	0.954	2.474
Time 2→10 min	O	0.475	0.639	1.821
Time 2→10 min	E50	1.676	2.300	1.722
Temperature 50→100°C	W	0.631	0.751	1.092
Temperature 50→100°C	NW	0.769	1.613	1.019
Temperature 50→100°C	G65	0.937	-0.007	1.410
Temperature 50→100°C	O	0.511	0.807	0.955

Table S12. Input matrix used for the time-trajectory PCA.

Code	Solvent	Temperature (°C)	Time (min)	TPC (mg GAE/L)	TFC (mg ECE/L)	DPPH (mM TE)
W	Water	100	2	397.829	249.575	1.035000
W	Water	100	4	453.910	329.180	1.144000
W	Water	100	6	804.476	503.175	1.176000
W	Water	100	10	860.691	518.572	1.199000
NW	Nanowater	100	2	222.168	180.257	1.007000
NW	Nanowater	100	4	310.452	194.120	1.112000
NW	Nanowater	100	6	539.604	289.867	1.180000
NW	Nanowater	100	10	573.487	412.050	1.240000
G65	65% glycerol-water	100	2	207.250	74.120	0.102100
G65	65% glycerol-water	100	4	219.250	99.850	0.150200
G65	65% glycerol-water	100	6	257.456	137.966	0.364913
G65	65% glycerol-water	100	10	395.160	143.600	0.567200
O	Rapeseed oil	100	2	294.533	179.687	0.134000
O	Rapeseed oil	100	4	323.287	182.753	0.254000
O	Rapeseed oil	100	6	391.209	191.853	0.356000
O	Rapeseed oil	100	10	409.243	279.910	0.473523
E50	50% ethanol-water	50	2	14.750	7.150	0.016400
E50	50% ethanol-water	50	4	24.500	7.120	0.024700
E50	50% ethanol-water	50	6	38.200	23.200	0.043100
E50	50% ethanol-water	50	10	47.120	35.200	0.054114

Variables were autoscaled before PCA. Matrix size: 20 conditions × 3 response variables.

Table S13. Input matrix used for the temperature-trajectory PCA.

Code	Solvent	Temperature (°C)	Time (min)	TPC (mg GAE/L)	TFC (mg ECE/L)	DPPH (mM TE)
W	Water	50.0	10	555.784	308.146	0.562300
W	Water	80.0		736.326	462.395	0.983400
W	Water	100.0		860.691	518.572	1.199000
NW	Nanowater	50.0		336.487	134.663	0.612000
NW	Nanowater	80.0		394.799	219.897	0.965200
NW	Nanowater	100.0		573.487	412.050	1.240000
G65	65% glycerol-water	50.0		206.365	144.250	0.213400
G65	65% glycerol-water	80.0		293.509	147.652	0.389200
G65	65% glycerol-water	100.0		395.160	143.600	0.567200
O	Rapeseed oil	50.0		287.200	160.020	0.244300
O	Rapeseed oil	80.0		397.500	280.200	0.321400
O	Rapeseed oil	100.0		409.243	279.910	0.473523

Variables were autoscaled before PCA. Matrix size: 12 conditions × 3 response variables. E50 was excluded because its temperature design was not directly comparable.

Table S14. CLR-PCA loadings for the six-marker essential-oil subcomposition.

Marker	PC1 loading	PC2 loading
Myrcene	−0.8627	−0.2378
p-Cymene	−0.0219	0.4271
γ-Terpinene	0.1303	0.0155
Linalool	0.2885	0.2631
Thymol	0.0803	0.3055
(E)-Caryophyllene	0.3855	−0.7735

PC1 and PC2 explained 72.8% and 17.4% of the compositional variance, respectively (90.2% cumulatively). Loadings were obtained from PCA of the reclosed six-component profiles after CLR transformation and mean-centering without additional autoscaling. The sign of a PCA axis is mathematically arbitrary; therefore, interpretation should focus on the relative signs and absolute magnitudes of the loadings within each component.

Table S15. Sensitivity of the integrated polar-functional index (IPFI) to the normalization set and variable weights.

Panel A. Baseline and alternative normalization/weighting scenarios.

Code	Baseline 1:1:1	NW excluded	TPC 2:1:1	TFC 1:2:1	DPPH 1:1:2
W	0.989	1.0	0.992	0.992	0.983
NW	0.809	—	0.771	0.805	0.853
G65	0.387	0.392	0.404	0.356	0.404
O	0.461	0.466	0.465	0.48	0.44
E50	0.055	0.055	0.055	0.058	0.052

Panel B. Equal-weight, two-response geometric means (the third response omitted).

Code	TPC + TFC	TPC + DPPH	TFC + DPPH
W	1.0	0.983	0.983
NW	0.728	0.816	0.891
G65	0.357	0.458	0.356
O	0.507	0.426	0.454
E50	0.061	0.049	0.054

All values were recomputed from the condition means given in Table S10. For each scenario, responses were normalized to the observed maximum in the relevant comparison set. For the NW-excluded scenario, normalization and ranking included only W, G65, O, and E50; no NW value is reported in that column. Alternative weights are in the order TPC:TFC:DPPH. No new experimental measurements were used. The order W > NW > O > G65 > E50 was preserved for the baseline, all three one-variable double-weight scenarios, and two of three two-response scenarios. When TFC was omitted (TPC + DPPH), G65 (0.458) exceeded O (0.426), whereas W and NW remained first and second, respectively. Excluding NW from normalization preserved the four-system order W > O > G65 > E50. These indices are descriptive, not inferential tests.

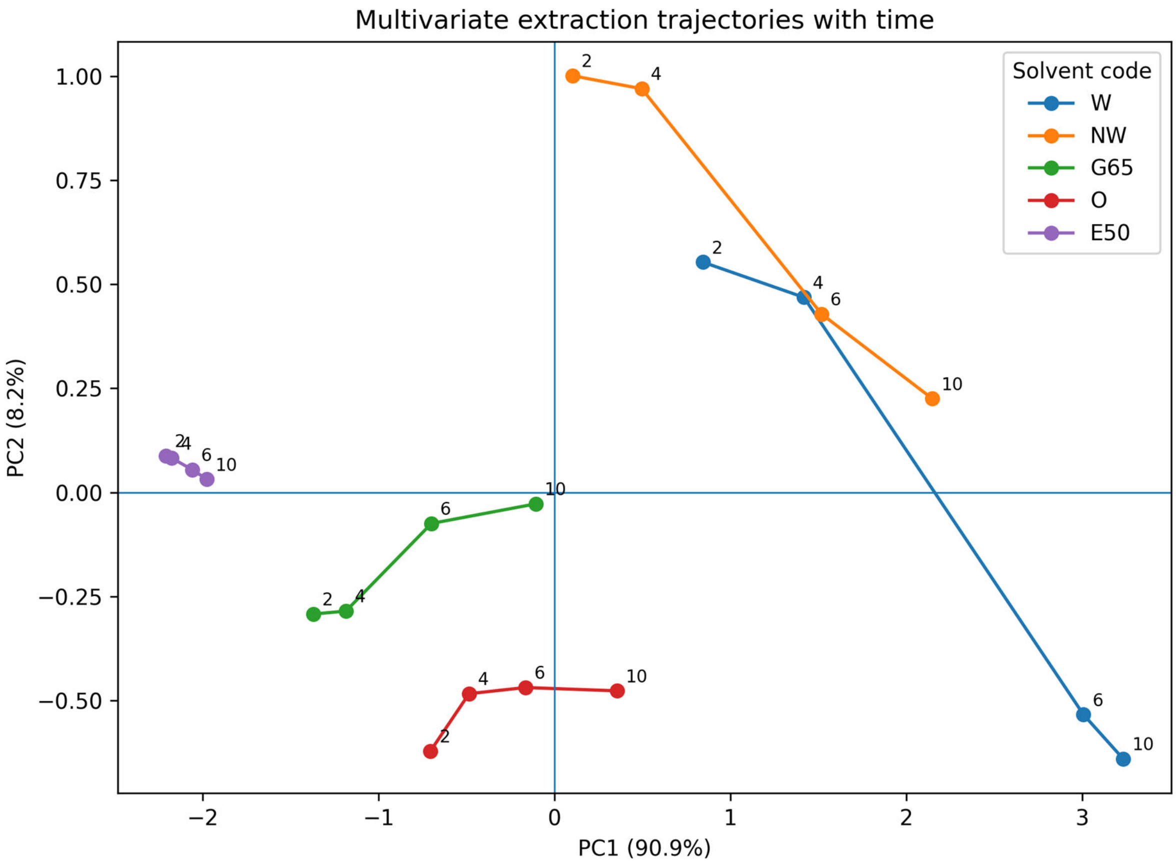

Figure S1. PCA trajectories of the combined TPC, TFC and DPPH response as extraction time increased from 2 to 10 min. Numbers next to the points denote extraction time (min). Variables were autoscaled before PCA. Solvent codes: W—water; NW—nanowater; G65—65% glycerol–water; O—rapeseed oil; E50—50% ethanol–water.

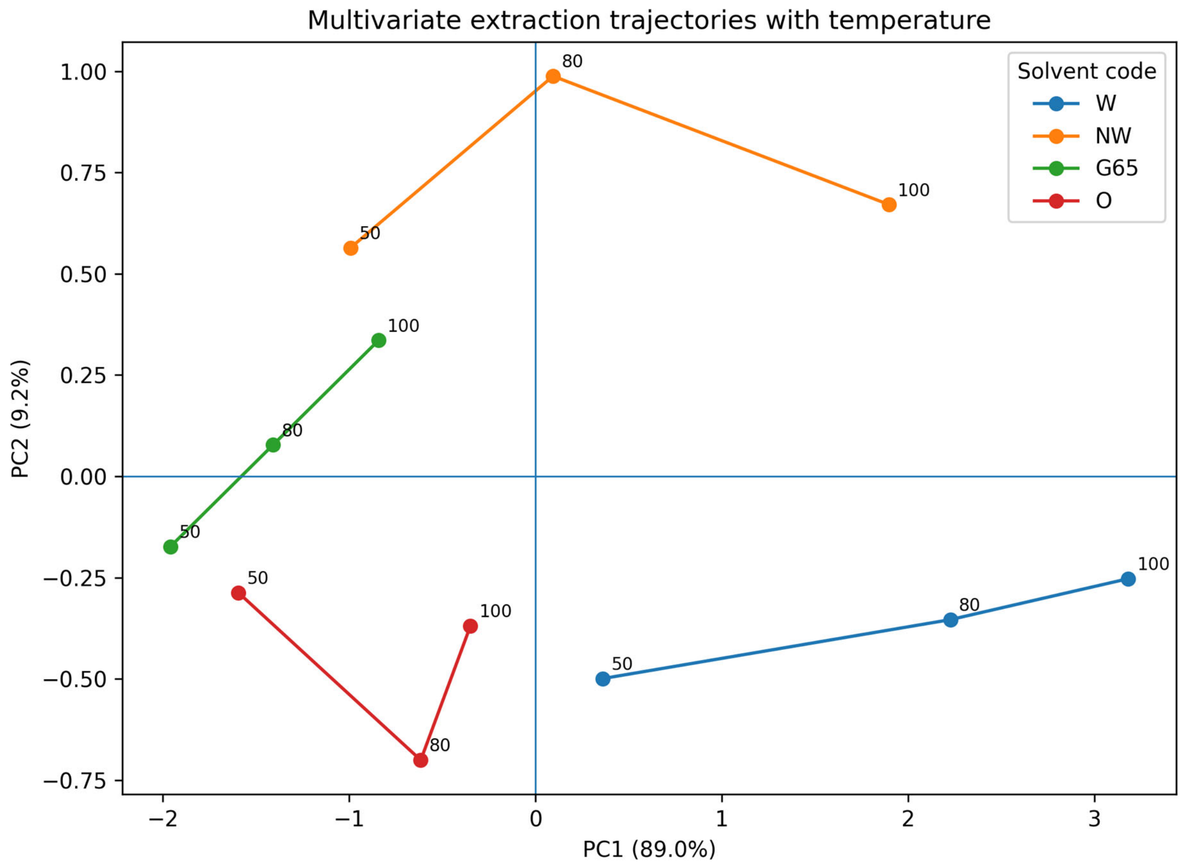

Figure S2. PCA trajectories of the combined TPC, TFC and DPPH response as the initial extraction temperature increased from 50 to 100°C at 10 min. Numbers next to the points denote temperature (°C). Variables were autoscaled before PCA. E50 was not included because its temperature scheme was not directly comparable.
